# Unsupervised deep clustering of high-resolution satellite imagery reveals phenotypes of urban development in Sub-Saharan Africa

**DOI:** 10.1016/j.scitotenv.2025.179739

**Published:** 2025-06-05

**Authors:** A Barbara Metzler, Ricky Nathvani, Viktoriia Sharmanska, Wenjia Bai, Simon Moulds, Nkechi Srodah Owoo, Iris Ekua Mensimah Fynn, Emily Muller, Esaie Dufitimana, Ghafi Kondi Akara, George Owusu, Samuel Agyei-Mensah, Majid Ezzati

**Affiliations:** 1 Department of Epidemiology and Biostatistics, School of Public Health, Imperial College London, London, UK; 2 MRC Centre for Environment and Health, Imperial College London, London, UK; 3 Department of Informatics, University of Sussex, UK; 4 Department of Computing, Imperial College London, London, UK; 5 Department of Brain Sciences, Imperial College London, London, UK; 6 School of Geosciences, University of Edinburgh, UK; 7 Department of Economics, University of Ghana, Legon, Accra, Ghana; 8 Department of Geography and Resources Development, University of Ghana, Legon, Accra, Ghana; 9 African Institute for Mathematical Sciences Research and Innovation Centre, Kigali, Rwanda; 10 Institute of Statistical, Social & Economic Research, University of Ghana, Legon, Accra, Ghana; 11 Department of Geography and Resource Development, University of Ghana, Legon, Accra, Ghana; 12 Regional Institute for Population Studies, University of Ghana, Legon, Accra, Ghana; 13 Abdul Latif Jameel Institute for Disease and Emergency Analytics, Imperial College London, London, UK; 14 Imperial Global Ghana Hub, Accra, Ghana

**Keywords:** Unsupervised deep learning, remote sensing, machine learning, DeepCluster, high-resolution satellite imagery, urban phenotypes, sub-Saharan Africa

## Abstract

Sub-Saharan Africa and other developing regions have urbanized extensively, leading to complex urban features with varying presence and types of roads, buildings and vegetation. We use a novel hierarchical deep learning framework and high-resolution satellite images to characterize multidimensional urban environments in multiple cities. Application of the model to images from Accra, Dakar, and Dar es Salaam identified areas with analogous patterns of building density, roads and vegetation. These included dense settlements within the metropolitan boundary (20-54% of urban area), peri-urban intermix of natural and built environment (21-44%), natural vegetation (9-13%) and agricultural land (8-15%). Kigali, with its mountainous geography and post-colonial expansion, exhibited unique urban characteristics including a sparser urban core (23%) and significant wildland-urban intermix (19% of vegetation). Other notable clusters were water (2% of area of Accra) and empty land (8-10% of Accra and Dakar). Our results demonstrate that unlabeled satellite images with unsupervised deep learning can be used for consistent and coherent near-real-time urban monitoring, particularly in regions where traditional data are scarce.

## 1 Introduction

Cities in the developing world are growing and changing rapidly. Cities and urbanization present opportunities to enhance the health and wellbeing of residents, as well as challenges on how to accommodate the growing population while minimizing adverse impacts on the local and global environment ^1^.

The environmental characteristics of a city arise from an interplay of regional environmental conditions (e.g., coastal, riverside, or mountainous; tropical or semi-arid; etc.), and how it has expanded and changed over time (e.g., patterns and density of roads and buildings.) ^2^. Therefore, cities with similar environmental conditions and/or historical trajectories can exhibit shared characteristics ^3^. For example, many cities in the United States have similarities in terms of sprawl and (poor) connectivity, which were influenced by automobile transport in the second half of the 20^th^ century ^4^. Cities in East and Southeast Asia have experienced substantial vertical growth, with the average building height rising over time ^5^. In contrast, cities in Sub-Saharan Africa (SSA), tend to have low-rise buildings with sharp spatial inequalities and increasing signs of urban sprawl ^6,7^. These differing development patterns raise the need to effectively monitor and guide sustainable urban growth in diverse settings, while safeguarding the intricate features that contribute to the distinctiveness as well as commonalities of cities. This challenge and need is becoming more relevant, due to the growing urban population and continued urbanization across the world, and especially in SSA which is the world’s fastest urbanizing region ^8^.

To support and evaluate policies for sustainable and healthy urban development, timely data on urban built and natural environments are needed. Data on various characteristics such as vegetation, roads, buildings and population settlements, are limited and infrequently gathered in low-income countries. The available data on these characteristics of cities have inconsistent spatial and temporal coverage and resolution, particularly across countries and regions. Consequently, tracking multiple urban characteristics is difficult, especially if comparisons across cities are made. Multi-city and multi-country studies on urban environments have focused on individual characteristics, such as the extent of cities ^9,10^, land use and land cover ^11–13^, including green and blue spaces ^9,14^, roads and connectivity ^10,15^, and population density ^16–18^. However, these features are interrelated and display complex patterns across different scales. Addressing this complexity, especially in multiple cities, requires data and methods that enable a holistic view of the urban environment both within and across individual cities.

Recent advancements in computer vision techniques, particularly unsupervised deep learning, offer promising approaches for analyzing complex urban environments from high-resolution satellite imagery. Unlike traditional supervised methods that require extensive labeled training data, unsupervised approaches can discover patterns without predefined categories, making them particularly valuable in data-scarce contexts. High-resolution satellite images can reveal fine-grained details (e.g., roads, rooftops, vegetation) while also enabling analyses of larger spatial configurations (e.g., neighborhoods, connectivity) ^19,20^.

In particular, unsupervised and self-supervised learning approaches provide a data-driven pathway for uncovering patterns in imagery without predefined labels, thereby mitigating the need for extensive ground-truth data, which can be scarce and costly in low-resource settings ^21,22^. By operating directly on visual inputs, unsupervised models can discover so-called “phenotypes” of the urban form—clusters that capture distinct combinations of built and natural characteristics—while allowing complex interrelationships (e.g., between vegetation, buildings, and roads) to emerge organically.

**Fig. 1:**
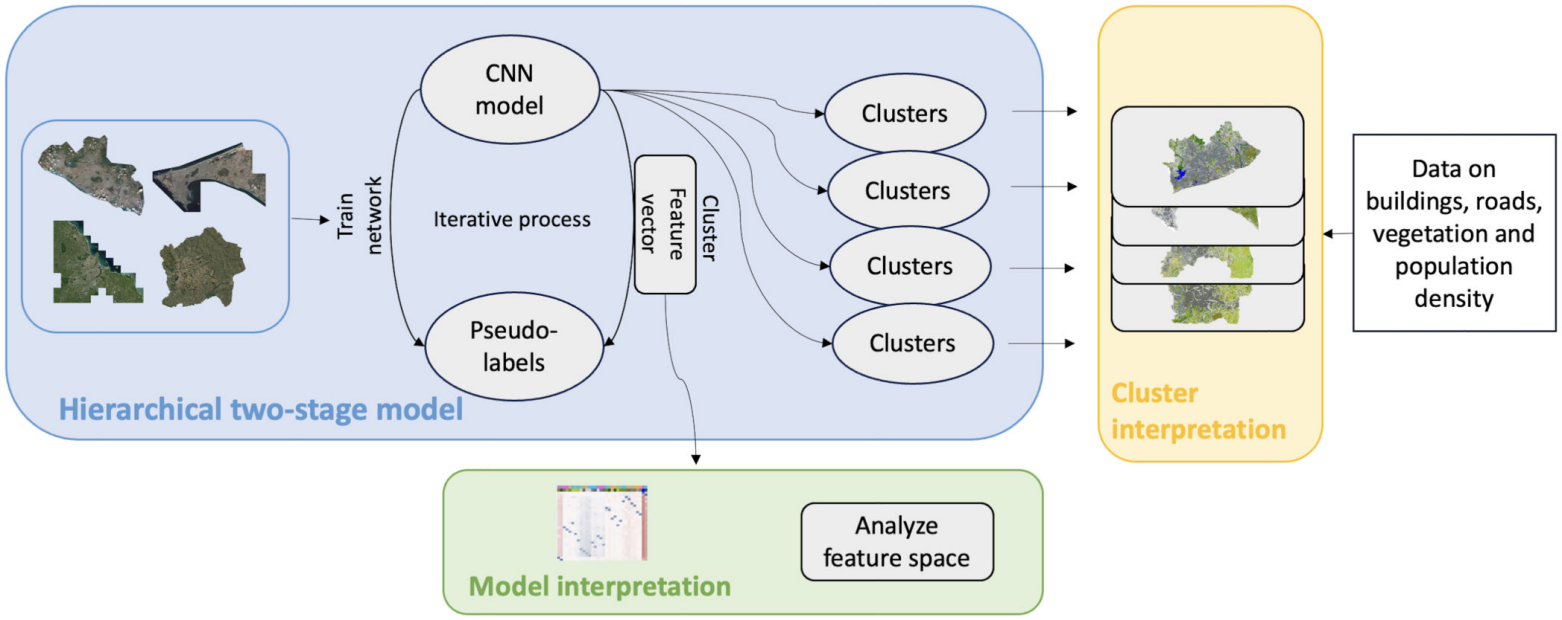
Overview of data management, analysis and interpretation. In the pre-processing step, the satellite images of Accra, Dakar, Dar es Salaam and Kigali were cropped into tiles. The image tiles were then fed into the end-to-end *DeepCluster* model to learn image representations. The extracted feature vector was clustered with k-means algorithm (K=8), separately for each of the four cities. The resultant clusters were interpreted with external data on buildings, roads, population density, water and vegetation. To understand how the model learns the image representations, we analyzed the clusters in the low-dimensional feature space.

Cities exhibit diversity in their environment, due to their geographic and environmental context, historical formation, economic development, and planning and policy decisions. Although single-city clustering approaches have demonstrated the potential of using satellite images and unsupervised deep learning to unravel complex urban patterns ^21^, addressing multiple cities together requires methods that accommodate both shared and city-specific urban features. A methodological challenge when applying unsupervised learning to multiple urban contexts is accounting for both commonalities and variations in environmental features across cities. For example, a clustering allocation of visual features that works in one city may over- or under-cluster visual features in another city with different socioeconomic or environmental characteristics. To address this challenge, we present and apply a novel hierarchical two-stage clustering approach to very high-resolution satellite images from four major SSA cities: Accra (Ghana), Dakar (Senegal), Dar es Salaam (Tanzania), and Kigali (Rwanda) (Table S1). First, we use a deep clustering framework^31^, where the convolutional neural network (CNN) learns shared image representations across cities. This shared representation leverages commonalities in urban structure—such as roads, rooftops, and vegetation—while retaining flexibility to capture city-specific nuances. Second, we perform city-specific clustering of learned representations to identify distinct cluster groups (or phenotypes) within each urban area. The resulting clusters thus balance comparability of visual features with the capacity to reveal cross-city diversity (Fig. 1).

The aim of this study is to develop and apply a hierarchical unsupervised deep learning framework to identify and compare patterns of urban form across four major Sub-Saharan African cities, using only high-resolution satellite imagery as input. We hypothesize the visual information captured by satellite images, when used with novel methods from machine learning and unaccompanied with any additional data, provide interpretable and practical insights into the spatial arrangement of built and natural environments in a way that is both coherent and consistent across cities. Therefore, such visually derived “phenotypes” could inform, and help track the impacts of, urban planning and policy choices. To interpret and validate the coherence, of these resultant clusters, we use demographic and environmental data not used during clustering. This step allows us to assess how well the purely visual clusters map onto known urban or socio-environmental characteristics—an important consideration for researchers and policymakers seeking insights beyond visual appearance. This auxiliary analysis demonstrates that our multi-city framework facilitates meaningful and actionable comparisons across cities while still identifying unique local phenotypes.

## 2 Data and methods

We applied an unsupervised clustering approach to very high-resolution satellite images from four sub-Saharan African cities and then interpreted the outcomes using external data on built and natural environments. The study cities were selected based on estimated and projected population change in the past and their location, and the similarity and diversity of their geography (East and West Africa), landscapes (coastal/inland) and climate zones (Table S1). We used the official administrative city boundaries to define the spatial extent of the cities included in the analysis; Each city boundary, except for Accra, refers to the city region, which is equivalent to administrative level 1. For Accra, we used the Greater Accra Metropolitan Area, since the city region (Greater Accra Region) includes large parts of rural Ghana. Fig. 1 provides an overview of the full workflow, including image pre-processing and analysis. The satellite imagery and environmental datasets are described in detail (section 2.1), followed by an explanation of the clustering framework (section 2.2). Finally, we describe how we linked external demographic and environmental data—excluded from the clustering process—to the derived urban clusters using a supervised classifier and SHAP analysis (section 2.3).

### 2.1 Data sources

#### 2.1.1 Satellite images

The satellite raster data was acquired from Maxar Technologies in GeoTIFF format. The spatial resolution of the images is 0.3 meters per pixel and captures three different bands (RGB). The high resolution of the satellite image allows for observing detailed urban characteristics such as building materials, e.g., cars, visual differences of concrete structures or metal sheet roofs (Fig. S1 in Supplementary Materials), while covering entire areas of the city and hence capturing the organization of these urban characteristics. Color balancing and orthorectification were applied to the images as part of commercial pre-processing. We cut the satellite images of all four cities into 256 x 256 pixel tiles, which is equivalent to about 75 m x 75 m on the ground and removed the tiles which covered the ocean. The choice of tile size is based on an in-depth cluster analysis of Accra ^21^ which showed that tiles of this size contain multiple features of urban form, e.g., houses and roads, such that it captures objects with their urban context and surroundings. Table S2 lists the number of tiles used for the analysis and further details about coverage and year of satellite image capture.

#### 2.1.2 Built environment, water, vegetation and population

We used four different datasets on urban characteristics (building area, building count, building orientation, NDVI, population density, distance to all roads, distance to major roads, length of major roads, length of all roads and average buildings size) in each city to interpret the image-based clusters. The vector and raster datasets capturing the urban characteristics are described in Table S3 and were not used in the unsupervised model.

### 2.2 Clustering methodology

#### 2.2.1 DeepCluster: Combined feature extraction and clustering

We employed *DeepCluster* ^23^, a method that merges the learning of image representations and clustering. In this process, a Convolutional Neural Network (CNN) extracts image representations from satellite image tiles. The CNN extracts visual features hierarchically, with features such as lines and edges in early layers and domain-specific features such as rooftops and trees in latter layers of the network, ultimately summarizing each image tile into 4096 numerical features. The 4096-dimensional image representations are compressed to 256 dimensions through a principal component analysis and are then normalized. These refined image representations are next supplied to a k-means clustering algorithm as stated in detail below. Each image tile is tagged with a cluster membership. These tags act as pseudo-labels that update the CNN’s weights. The model then refines both clustering and classification with each training cycle, generating new pseudo-labels after every epoch. The entire approach is purely data-driven and not influenced by the presence or absence of labeled data. *DeepCluster* is an efficient and resource-flexible method for understanding inherent data groupings, making it less computationally intensive than frameworks like contrastive learning ^24^. We chose *DeepCluster* over alternatives like autoencoders and Principal Component Analysis (PCA) because it can capture complex (nonlinear) patterns within images through joint feature learning and clustering, and can be trained and scaled across large datasets.

We used the CNN architecture VGG-16 ^25^ that had been pre-trained on the ImageNet dataset for feature extraction. CNNs trained on large datasets like ImageNet are commonly used to leverage low- and mid-level features that generalize well across tasks, even when the target domain differs from natural images ^26,27^. This approach improves and accelerates learning, particularly in unsupervised settings where there are no external labels ^28^. We selected VGG-16 for its architectural simplicity and demonstrated effectiveness in our previous single-city analysis ^21^, which supported its suitability for image representation learning and unsupervised clustering. While more recent architectures like Vision Transformers have shown strong performance on large-scale benchmarks, they typically require substantially more training data and compute resources. We retained the hyperparameters from the original *DeepCluster* paper, with adjustments in the epoch number and learning rate as needed for finding the optimal convergence of the algorithm. Details on how we chose the learning rate are available in the Supplementary Materials (Table S4).

We set a high initial cluster number (k) in *DeepCluster* to help the model learn a wide range of visual features across the four cities. The choice of the hyperparameter k in the k-means clustering part of the algorithm is distinct from the final number of clusters (K); rather, k influences how the algorithm learns low- and mid-level image representations. A large hyperparameter k is better suited for feature learning ^23,29^ even if for interpretation we prefer a smaller number of clusters as further explained in section 2.2.2. Subsequently, we extracted the image representation (n x 4096 vector, where n is the number of tiles) from the second last layer and split the representations by city. We then clustered the tiles, based on their image representations, with a k-means clustering algorithm with a K of 8 clusters as stated in section 2.2.2 and 2.2.3, implemented with the *scikit-learn* package ^30^. The entire training process, spanning 20 epochs, took around 72-76 hours for all cities combined. Training beyond this did not bring significant alterations to the clusters. The model was trained using 3 RTX6000 GPUs with a total RAM memory of 250GB.

#### 2.2.2 Hierarchical two-stage clustering

**Fig. 2:**
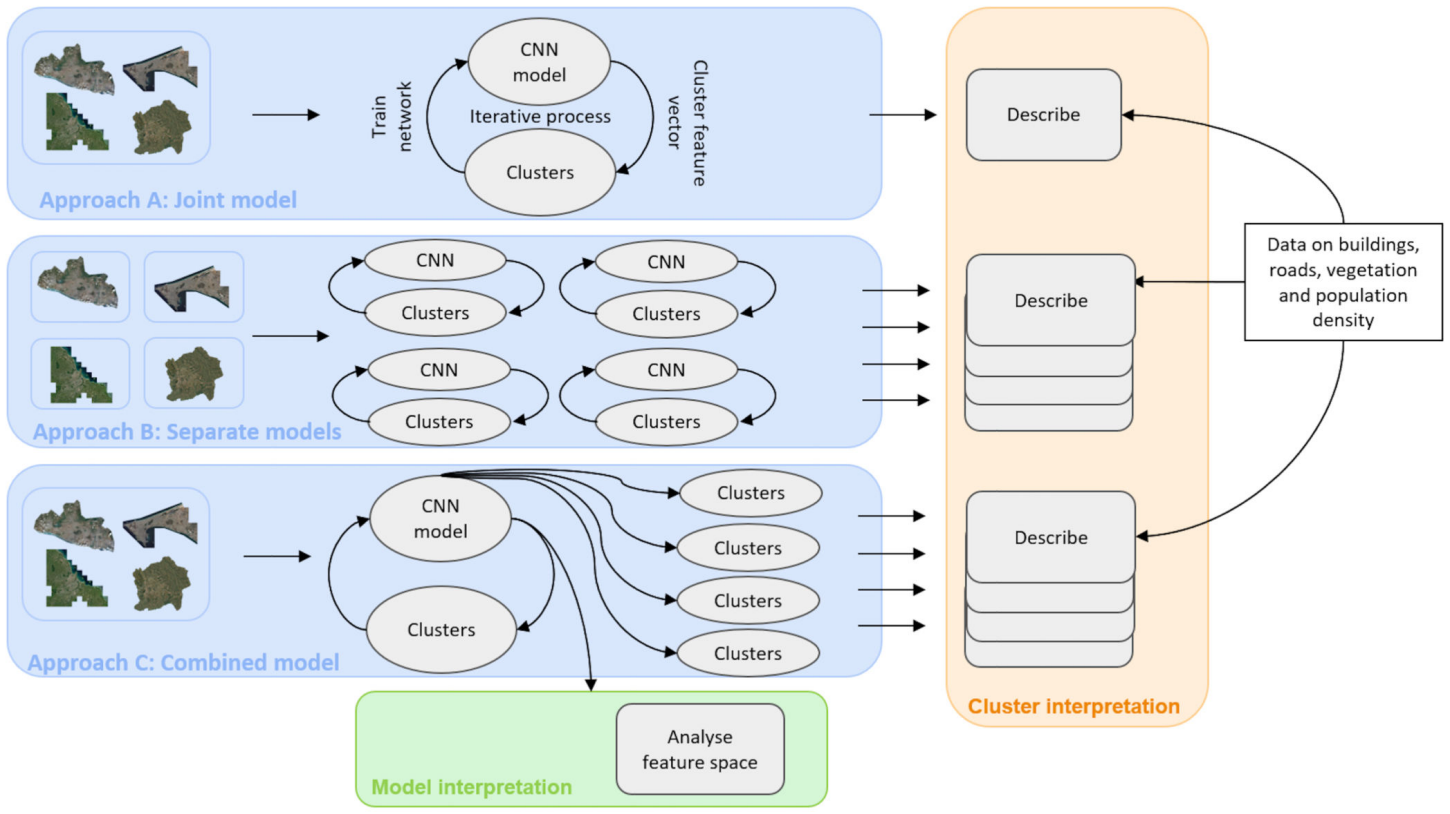
Overview of tested feature extraction and clustering approaches. In Approach A, the image representations are learned and clustered in one step for all cities together. In Approach B, the image representations are learned and clustered together for each city separately. Finally, in Approach C, the image representations are learned together, however, clustered individually per city. This approach allows for cluster comparisons in the feature space.

We chose our two-stage clustering approach on the basis of a preliminary analyses, in which we tested three candidate approaches (Fig. 2). As stated above, it is possible to modify the *DeepCluster* algorithm so that the choice of the hyperparameter k in the first step (feature extraction), which influences how the algorithm learns distinctive image representations, is distinct from the final number of clusters (K). Both steps can be included in a single pipeline or separately. We took advantage of this possibility to probe the performance of the various choices along the city-specific and joint spectrum to cluster the images of multiple cities. The three different approaches are as follows:

- Approach A: Joint one-stage model: Joint learning of image representations and clustering of all cities together.
- Approach B: Separate one-stage models: Separate learning of image representations and clustering of each city one at a time.
- Approach C: Hierarchical two-stage model: Joint learning of image representations across all cities and separate clustering by city.

In Approaches A and B, learning of image representations and clustering are performed in one step ^21^. Approach C includes two steps, where image representations of all the cities are learned together and subsequently clustered by city. The three approaches implicitly have different assumptions about urban features and clusters. Approach A assumes that the cities share similar environmental features, such that we find clusters that are shared between cities and hence the clustering is done together. When the analysis is done for each of the cities separately, as in Approach B, it is assumed there is no benefit to sharing information across cities during the learning of image representations and clustering because the inter-city variations are very high. Finally, the hierarchical two-stage approach, Approach C assumes that intercity similarities are useful for learning of image representations, but it is important to cluster cities separately to avoid missing any city-specific intricacies due to different distributions of urban land use. For example, the algorithm benefits from being fed a large variety of buildings across different landscapes to learn image representations, irrespective of their classification as part of densely populated areas or not. This last approach shares some similarities with transfer learning, where knowledge gained from training on one task is used to enhance the learning and performance of a different but related task.

To compare the three different approaches, we measured the homogeneity of each cluster in relation to their respective urban characteristics, because by design clustering should group more similar image tiles together. The urban characteristics used were building area, building count, building orientation, NDVI, population density, distance to all roads, distance to major roads, length of major roads, length of all roads and average buildings size. We computed the deviation of each urban characteristic per tile and cluster, relative to the median of its assigned cluster, referred to as the Median Absolute Deviation (MedAD). The MedAD measure is calculated as follows:

$$MedAD=median(|y_{i}-\hat{y}|)$$

where *y*_*i*_ represents the actual urban characteristic for each tile and ŷ represents median urban characteristic for each cluster. The overall sum of all MedAD scores across all urban characteristics, the Median Absolute Deviation (MAD) score, is defined as follows:

$$MAD\text{=Σ}median\text{(|}y_{\text{i}}–\hat{y}\text{|)}$$

We used the MAD score to summarize variability across urban characteristics for different cluster approaches. A cluster with highly variable urban characteristics would have a higher MAD than a cluster whose tiles are closer to the overall median of a certain urban characteristic (e.g., NDVI or building count) specific to that cluster.

Table 1 compares the MAD values among the above three approaches and calculated on a city basis, as well as across cities. Approach C achieved the lowest MAD scores for Dakar, Dar es Salaam and Kigali, and these scores were closely followed by those of Approach B for all three cities. Approach B achieved the lowest MAD for Accra, closely followed by Approach C. Approach A (with K=8) attained the highest scores across cities. We further calculated the MAD across all cities, where the hierarchical approach (Approach C) obtained the lowest MAD scores followed very closely by the separate approach (Approach A), with only a difference of 0.2474 in MAD.

In terms of results, the joint one-stage approach (Approach A) produced results that were difficult to interpret because it split off Accra from the other cities and further clustered tiles together that were not visually similar. The separate model approach (Approach B) and the two-stage approach (Approach C) could identify city-specific and shared clusters relevant for urban planning and policy-making. However, the process of matching clusters between cities and retraining the network for each city in Approach B was time-intensive, and at times inconclusive.

Approach C effectively identified shared features between cities while preserving city-specific intricacies. Although some specificity was lost, likely due to underrepresentation in the combined datasets. This approach enhanced comparability between cities, and allowed for more specific clusters to emerge through learning the image representations across cities. For example, in Accra, Dakar and Dar es Salaam, Approach C picked up densely populated areas with specific building orientations that in Approach B were mostly clustered together. Among all approaches, Approach C was the most time- and computation efficient, particularly when exploring different values of K, as the computationally intensive feature learning is performed only once, and the CNN does not require retraining for subsequent analyses.

In summary, Approach C struck a balance between computational efficiency and the interpretability of features across cities while preserving city-specific intricacies. Moreover, Approach C identified clusters with minimal deviation in urban characteristics, as indicated by the MAD score. Given these advantages, we used the hierarchical approach in our analysis.

#### 2.2.3 Cluster number (K)

As stated earlier in section 2.2.1, we selected the number of clusters as K=8 through a visual examination of results and an initial set of experiments. This number balances cluster separation and the level of detail needed for an intuitive classification of the urban environment, as seen in sensitivity analyses in sing-city analysis^21^. We kept the number of clusters in all approaches the same, such that we could make comparisons between cities and approaches. Internal analyses using the silhouette score and the Calinski-Harabasz criterion, which evaluate cluster cohesion and separation for different K values, supported the choice of K=8, as detailed in Supplementary Materials. However, neither method provided a unique and definitive choice for the best K, as shown in Fig. S2. Experiments based on cluster number also showed that lower cluster numbers usually distinguished between built and natural environments, and increasing K divided these categories into more detailed subcategories.

### 2.3 Built and natural environment and demographic characteristics and predictors of clusters

We used the data on the buildings and roads, water, vegetation, and population, not used in the clustering process, to quantify the characteristics of the clusters that were formed based on image data alone. Additionally, we used the machine learning classifier XGBoost ^31^ to quantify which environmental and demographic variables, individually and collectively, characterize the image-based clusters. This decision-tree-based method identifies which environmental and demographic variables predict cluster membership. It has the practical advantage of being able to accommodate missing values ^32^, such as image tiles that have no buildings and therefore no average building size and orientation. To measure which environmental and demographic variables are important for predicting image tiles’ membership to different clusters, we used the fitted classifier to generate SHAP ^33^ values. The SHAP values are summary measures of the importance of each environmental and demographic variable for each cluster as well as across all clusters, in an additive manner. We applied XGBoost on a city-by-city basis to focus on the environmental features of each city that relate to cluster formation, rather than focusing on development of the image representations across multiple cities.

We split the dataset (tiles) for each city into 70% for training and 30% for testing. We used a stratified splitting approach to ensure proportional representation of all clusters in the evaluation. The gradient boosting classifier, XGBoost, was fine-tuned with a 5-fold cross-validation method with classification accuracy as a score. We used the Hyperopt library ^34^, which uses Bayesian optimization for parameter tuning to find the optimal hyperparameters. The moderate to high accuracy levels (Table 2) indicate that the environmental and demographic variables incorporated into the SHAP analysis might only encompass a subset of the visible features. Other potential visual indicators, for which we did not have urban data, could include aspects like vehicles, distinct architectural features, specific vegetation types and varied terrains ^35^.

## 3 Results

### 3.1 Clusters in four cities in Africa

Fig. S3 in Supplementary Materials shows the clusters plotted on the city maps. We named and categorized the resulting clusters into super-groups based on the type of environment that they represent (Table 3), including: *Vegetation, Agriculture, Empty land, Water, Densely populated areas*, and *Other mixed environments* which contain both the natural and the built environment. Most super-groups were represented in all cities, except *Water, Agriculture* and *Empty land*. The *Water* cluster was only picked up in Accra (Fig. S3), despite Dakar having several lakes within the city boundary. The larger size of Accra’s water bodies may have led the model to classify them as a distinct cluster. In contrast, the water bodies in Dakar were included in a cluster that also encompassed sand and empty land, possibly due to their shared feature of displaying homogeneous surfaces in uniform color hues without any buildings or structures. The decision by the model not to segregate the water bodies in Dakar may also be attributed to the distinct colors exhibited by these bodies, like Lake Retba’s distinct pink hue in the satellite images which contrasts with other water bodies that had a vivid green color in the images. Accra was also the only city that did not capture the super-group *Agriculture. Empty land* was only picked up in Accra and Dakar.

Within super-groups, some cities had one cluster, whereas others had multiple. For example, we identified one *Densely populated area* cluster in Kigali, whereas in the other three cities, there were multiple *Densely populated area* clusters that differed in their building orientation (Fig. S4) and had differences in building density. Similarly, the approach captured a range of *Vegetation* clusters, ranging from shrubs to dark dense vegetation (Fig. S5). Dar es Salaam and Kigali, which are comparatively green, had three clusters that ranged from lush and dense vegetation to shrubs and grass, whereas Dakar, the city with the lowest levels of rainfall in our study (Table S1), had two *Vegetation* clusters that captured shrub-like vegetation which were not as lush as the *Dark dense vegetation* clusters in the other cities. The *Vegetation* clusters differed from the *Agriculture* clusters, with the latter presenting organized patterns in vegetation and crops which represent human-made monoculture farming (Fig. S6), demonstrating the method’s ability to use the visual information in satellite images for segmenting a city’s environment into interpretable clusters. We also identified clusters without any vegetation or built structures, including empty or sandy land in Accra and Dakar, and a cluster capturing water in Accra. Finally, the approach captured *Other mixed environments*, with elements of both the natural and the built environment, and with varying levels of buildings, roads and vegetation (Fig. S7). In Kigali, a second cluster fell into this category, characterized by moderate population density and typically situated on sloped terrain.

### 3.2 Cluster interpretation with variable prediction

As stated in section 2.3, we quantitatively evaluated what features of urban form, environment and population are most represented in the visually identified clusters, we trained a machine learning classifier to predict cluster membership using environmental and demographic variables not used in clustering, as described in section 2 Data and methods. The SHapley Additive exPlanations (SHAP) values are summary measures of the importance of each environmental and demographic variable for each cluster as well as across all clusters, in an additive manner ^41^.

**Fig. 3:**
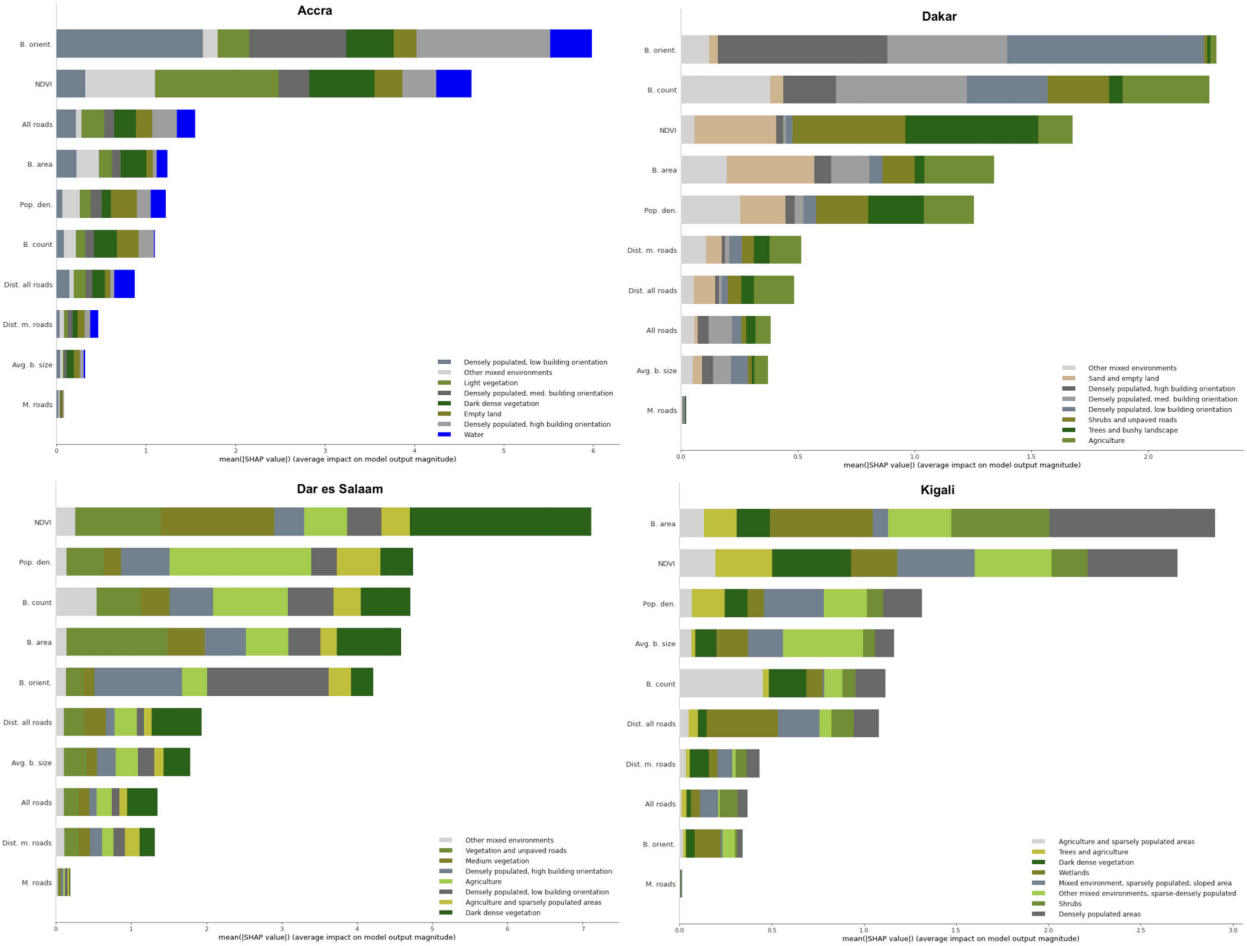
Built and natural environment and demographic variables as predictors of membership in image-based clusters. SHapley Additive exPlanations (SHAP) summary for Accra (Fig. 3A), Dakar (Fig. 3B), Dar es Salaam (Fig. 3C) and Kigali (Fig. 3D). The figure shows SHAP ^41^ values, obtained by fitting a XGBoost classifier to predict cluster membership by environmental and demographic variables per city. The SHAP value for each variable indicates its predictive power for assignment to various clusters, and hence identify the measures of urban form (buildings and roads), environment (water and vegetation), and population that differentiate clusters that were generated based on images alone. The mean SHAP values from the XGBoost classifier were calculated for each environmental and demographic variable as described in s 4.1.2. The total length of each bar, which is the mean absolute SHAP value, represents the overall importance of each variable for predicting cluster membership, and the different colors represent the importance for assignment to each cluster. NDVI: Normalized Difference Vegetation Index.

The SHAP results (Fig. 3) indicate that building information (building area, count and orientation) and Normalized Difference Vegetation Index (NDVI; an indicator of vegetation in a satellite image based on spectral absorption of light; range: −1.0 to 1.0) were important external predictors for cluster membership in all cities. Building orientation, which is defined as the deviation of orientation from cardinal directions, was particularly important in Accra and Dakar, and especially in predicting the *Densely populated areas* clusters. NDVI emerged as a significant predictor in all cities, especially for clusters associated with *Vegetation* and *Agriculture*, which were themselves distinguished based on how structured the green landcover was. Building count and area were also influential, with building count being significant for the built environment (very high building count) and *Agriculture* and *Vegetation* clusters (very low building count). In Dar es Salaam, NDVI and population density were the primary variables that predicted cluster membership, closely followed by building count and area. Similarly, in Kigali, NDVI played a crucial role in predicting cluster assignment, alongside population density and average building size. Overall, information on roads emerged as less predictive of cluster membership than other variables across the studied cities, including for the *Other mixed environments* clusters that capture a high number of roads. This may have occurred because building count and area were positively correlated with number of roads in all cities, and hence explain some of the cluster assignments that may have otherwise been predicted by roads.

### 3.3 Clusters in the feature space

**Fig. 4:**
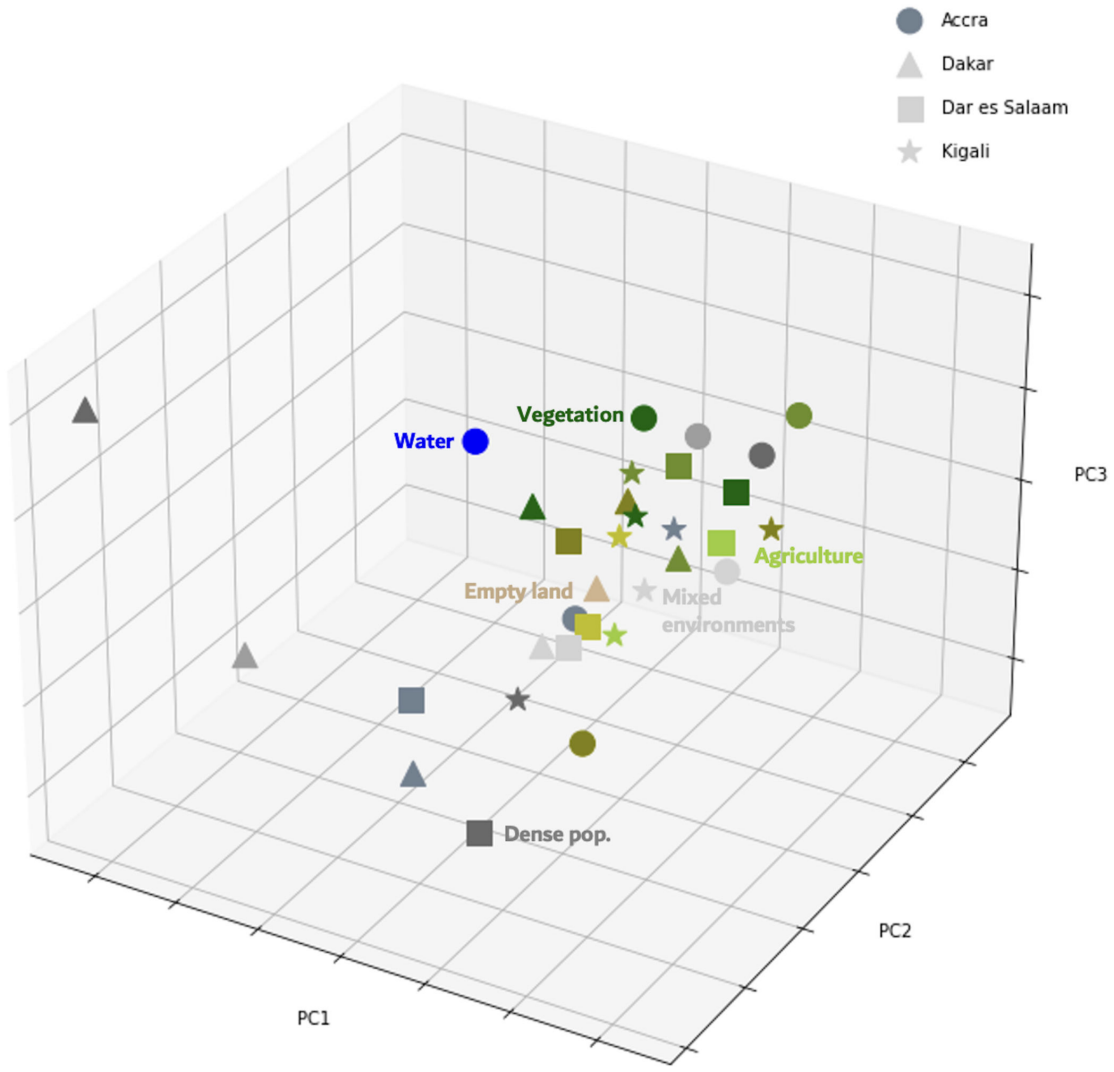
Cluster centroids seen in the CNN-extracted feature space. Cluster centroids plotted on the first, second and third principal component (PC) of the feature space. The colors of the points refer to the maps in Fig. S3 and Fig. S10, and the clusters are named based on their super-cluster category.

**Fig. 5:**
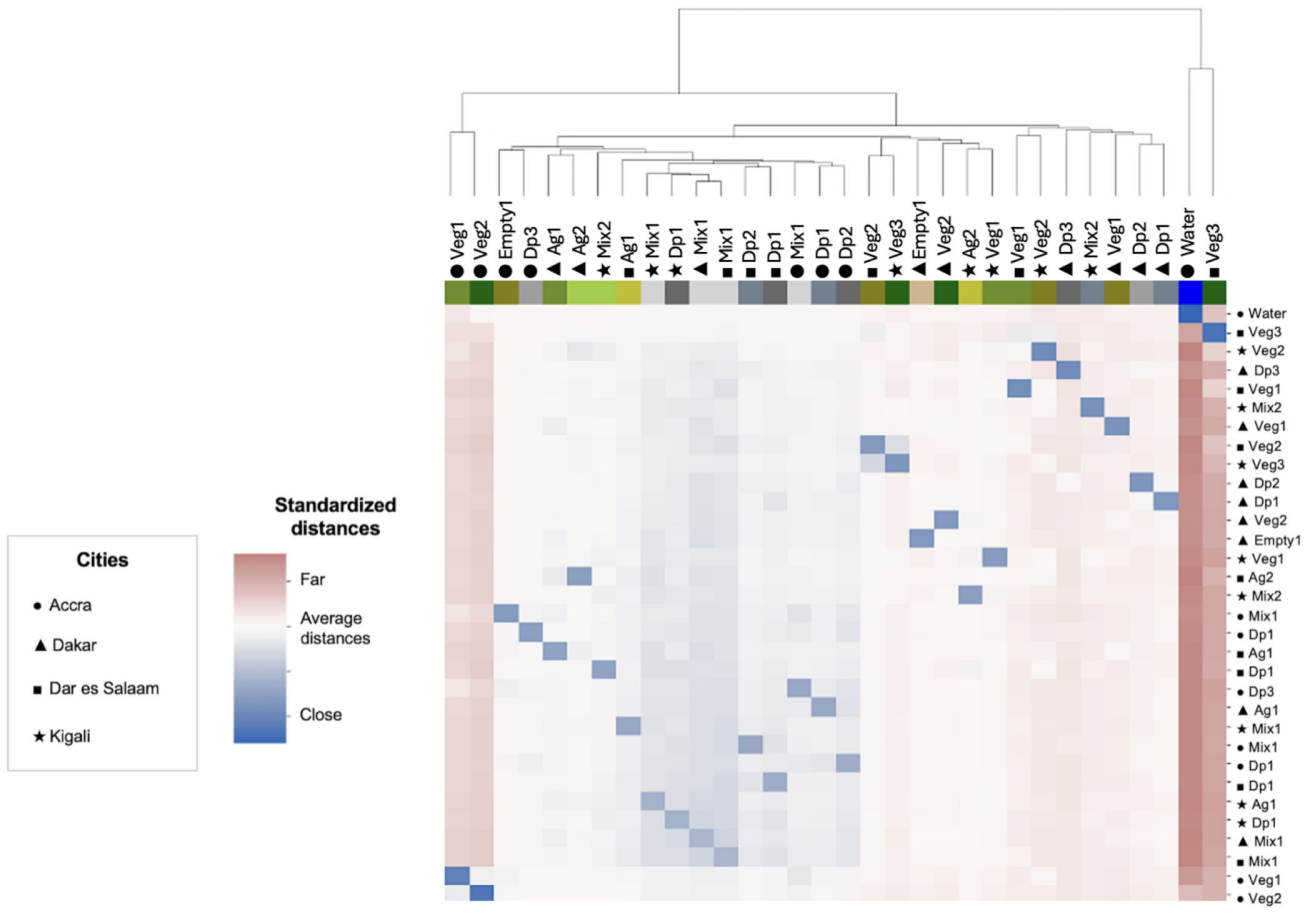
Inter-cluster distances with z-score normalization. The clusters are ordered by distance between cluster centroids, with the dendrogram indicating cluster distances (Euclidean distance) in the feature space. The color scale ranges from cool (blue) to warm (red), indicating close proximity (blue) and greater distances (red) between clusters in the feature space. Clusters within the same super-group – *Vegetation, Agriculture, Densely populated areas* and *Other mixed environment* clusters – were more closely situated in the feature space than those in different super-groups. See Table 3 for cluster names and typologies.

As stated earlier, we used a hierarchical two-stage model that initially learns image representations through a CNN, and then clusters the extracted image representations for each city. To understand what image representations are learned and used for clustering, we visualized cluster centroids for each cluster in the lower-dimensional feature space. The learned image representations were subsequently dimension reduced as stated in section 2 for computational efficiency and visual presentation via PCA (Fig. 4). We found that city-specific clusters within the same super-group – *Vegetation, Agriculture, Densely populated areas* and *Other mixed environment* clusters – tended to be closely situated in the feature space. This alignment is further supported by Fig. 5 which illustrates inter-cluster distances.

In all cities, there was a large separation between *Densely populated areas* and other clusters (Fig. 4, Fig. 5). The mixed environment clusters (*Other mixed environments*) were generally close to one another, as were many *Densely populated areas* clusters (*Dense pop*). The exception to this pattern was the *Densely populated areas* cluster of Kigali, which was closer to the *Other mixed environment* clusters for other cities. Examination of satellite image tiles shows that the *Densely populated areas* cluster in Kigali, while being the most densely populated within Kigali itself, were less densely populated compared to analogous clusters in other cities. In fact, the tiles showed more of an intermix between natural environments and buildings.

As shown in Fig. 5, some *Densely populated areas* and *Vegetation* clusters in Dakar were notably more distant from the clusters of the other cities. This is likely due to the dry and sandy nature of the city, which manifests in different color ranges in vegetation and built environment compared to the other cities, and the boxy concrete structures in Dakar which are different from those of other cities (Fig. S8). In addition, Dakar does not have as many metal roof sheets, which are commonly seen in SSA cities ^36^, as the other cities thus presenting different color ranges compared to the other cities. The clusters of Accra and Dakar were, on average, the furthest apart in the feature space, with the other cities’ clusters positioned in between. This distinctiveness is largely attributed to Accra’s natural landscapes, such as the *Water* cluster and the *Dark dense vegetation* cluster capturing very homogenous green features (Fig. S9). The distance of Accra in the feature space, however, could also be due to the commercial preprocessing of the images, since the Accra images were captured and processed about 2-3 years before those of the other three cities (Table S2).

### 3.4 Cluster size and arrangement within cities

We found similarities in cluster locations and spatial arrangements in the study cities, particularly among Accra, Dakar, and Dar es Salaam. These similarities were evident despite the different city sizes, and could be attributed to their historical or environmental similarities (Table S1). The analysis identified *Densely populated area* clusters in all cities, typically aligning with metropolitan centers hosting markets and transport hubs (Fig. S3). Fig. S10 shows the proportions of cluster membership per city. Accra had the largest percentage of areas with high building density, with over 50% of its tiles being medium to highly populated. The other three cities each had 20-30% of their areas with high population density. In all cities, these *Densely populated areas* clusters gradually thinned out as they extended from the city center. However, pockets of dense populations were also present in regions removed from the urban core, typically clustered around major roads or areas of trade. Visual analysis of satellite image tiles and analysis with external data confirmed these as informal settlements, characterized by high population density, small buildings, and corrugated iron roofs (Fig. S8). In cities with multiple *Densely populated areas* clusters, namely Accra, Dakar and Dar es Salaam, the main discriminator between clusters was building orientation with respect to cardinal directions (Fig. S4). Kigali, which is a less densely populated city, had only one *Densely populated areas* cluster, with no discrimination related to building orientation. The lack of neighborhoods with systematic building orientation in Kigali can be attributed to the city’s varying elevation and geographic nuances.

All four cities had at least two *Vegetation* clusters and Dakar, Dar es Salaam and Kigali had at least one *Agriculture* cluster. The distribution of *Vegetation* and *Agriculture* clusters was similar across Dakar, Dar es Salaam, and Kigali. The size and arrangement of the *Vegetation* and *Agriculture* clusters varied depending on the cities’ climate and boundaries. Within-city green space was more common in tropical climate zones, especially in Kigali and Dar es Salaam, which contained a significant proportion of natural vegetation and agricultural land or cropland. Kigali had the highest percentage of *Vegetation* and *Agriculture* clusters, making up 43% of the city. Dakar and Dar es Salaam both have *Vegetation* and *Agriculture* clusters that made up about 30% of the city, and Accra showed the lowest coverage at 13%. In all cities, the majority of the *Vegetation and Agriculture* clusters were located outside the dense city center and next to peri-urban areas and sparsely populated areas. Nonetheless, the *Vegetation* clusters also captured urban green patches, and, in some cases, urban agriculture spread through the metropolitan areas.

In each city, one or two clusters captured areas with sparse to moderate building density, revealing a mix of built and natural environments, often including roads (*Sparse-moderately populated areas* cluster or other clusters that fall in the *Other mixed environments* super-group). This phenotype was the largest cluster (in Dakar, Dar es Salaam and Kigali), alone or along with a *Densely populated areas* cluster, in all cities (Fig. S10). Tiles assigned to this cluster were spread across the city and often represented the transition between the more homogeneous clusters, such as *Vegetation* and *Densely populated areas*.

## 4 Discussion

### 4.1 Formation of clusters

A city’s environment depends both on its specific parts, and their natural and built environmental features, and on how these parts are arranged in relation to one another. The clusters captured by our hierarchical two-stage clustering method, and their arrangements, have emerged from the interplay between the local environment and the historical and recent city expansion and planning.

A key finding is the influence of colonial urban planning and development that have led to similar phenotypes and spatial arrangements in Accra, Dakar, and Dar es Salaam. Specifically, in the three coastal cities, and especially in Accra and Dakar, this influence manifests as populated neighborhoods, which exhibit grid-like road structures ^37^. These areas were captured by the *Densely populated areas* clusters that identified rows of buildings aligned in a specific way (low, medium or high building orientation; Fig. S4). Such uniform patterns diminished in the peri-urban zones in all cities. Peri-urban areas are more influenced by contemporary urban planning and expansion, and which display more organic, unstructured road and building alignments, and hence have a larger intermix of the clusters, capturing both densely populated areas with specific building orientations as well as moderate density and mixed land use. The more organic and sparser arrangement of buildings in the peri-urban areas have emerged in more recent urban growth and sprawl ^6,7^. Kigali’s *Densely populated areas* did not exhibit specific patterns in building orientation. This may be attributed to the Belgian colonial approach that often discouraged urbanization ^38^ and the relatively recent establishment and growth of the city ^39,40^.

Architectural differences related to both historical and recent building sizes, forms, and materials also distinguished clusters across specific cities. For instance, Dakar’s cubic concrete buildings with flat roofs ^38^ contrast with the metal sheet and slate roofing prevalent in Accra and many East African cities ^41^. These distinctions are evident in the feature-space distances (Fig. 4, Fig. 5). Although the *Densely populated areas* clusters appear relatively similar overall, Dakar stands out as the most distant from the other cities, likely due to its characteristic of concrete flat roofs and its unique color signatures in satellite imagery (Table S1).

The impact of climate and geography, and its interaction with more recent urban expansion and development, is also evident in the formation of clusters, especially in the presence or absence of green spaces. Dar es Salaam and Kigali have green patches within them (Fig. S3), while Accra and Dakar lack extensive green spaces, partly due to interplay of their drier climate with historical and recent urban development, which favored expansion of housing for their growing populations ^42,43^. The vegetation within Kigali and Dar es Salaam includes natural green spaces with mixed plant species, as well as agricultural zones characterized by repetitive patterns of homogeneous crops. An intermix of *Agriculture* and *Densely populated areas* clusters highlight the metro-rural boundaries, particularly noticeable in Dar es Salaam and Kigali ^44–46^. Kigali’s urban layout is influenced by its diverse topography (e.g. steeper slopes), which further adds to a lower building density relative to other studied cities ^47,48^. As a result of this, Kigali is the only city with just one *Densely populated areas* cluster, unseparated by building orientation.

### 4.2 Applications for tracking sustainable urban development

Our findings can support sustainable urban development, for example in terms of the distinction in features of the *Densely populated areas* and *Other mixed environments* clusters which together capture most of the built-up areas and the paved and unpaved roads. The *Densely populated areas* clusters were present both in the central core and at farther distances from the dense city center ^6,7^, and were especially concentrated near primary commercial harbors in the three coastal cities. The *Other mixed environments* further encompass industrial and commercial areas, predominantly situated near the harbor and typically within or adjacent to city centers, exerting added pressure on the already taxed road network. Both clusters capture residential areas, but informal markets and street-level commerce are more prominent in the *Densely populated areas*, particularly in informal settlements and high-traffic neighborhoods near transport hubs. Despite often being associated with lower economic status compared to other parts of the city, their strategic position near transportation and commerce hubs makes them centers for both formal and informal trade including marketplaces ^49^. To mitigate the increased noise and air pollution and congestion that often accompany these features ^50,51^, environmental regulations and technologies such as lower-emission transport options are needed ^52,53^. Further, as African cities experience additional hot days due to global and regional climate change, the density of these areas and their limited vegetation can increase human exposure to heat ^54,55^, requiring sustainable cooling strategies such as green space and shading. Similarly, building features such as material and building orientation impact indoor heat ^56–58^. A comparative analysis across cities can show the characteristics and locations of such economic and environmental hotspots and better inform urban planning to improve their vitality while protecting them from environmental consequences of density. For example, although Accra has decentralized some of the congestion by relocating its primary shipping operations to Tema Port, both Dakar and Dar es Salaam maintain their primary cargo facilities within their central metropolitan area. Additionally, in Dar es Salaam, industrial lands are mainly distributed along the industrial corridor that encircles the city’s railway infrastructure and reaches into the city center ^45^. Kigali’s master plan, in contrast, includes the establishment of a special economic zone in the peri-urban area near the airport, with the aim of redirecting trade and reducing congestion ^59^. More recently, there has been a trend towards taller structures in these areas, contrasting with previous urban sprawl patterns observed in major African cities ^6^. Vertical densification and renewal, as currently planned in Kigali, can increase economic productivity, while also displacing their current residents unless accompanied with appropriate housing in the same or nearby locations as a part of redevelopment ^59,60^. It will also likely change the visual characteristics of these areas and hence can be measured and monitored through the approach that we presented.

Our method went beyond traditional greenness measures like NDVI, and successfully identified various types of vegetation. In particular, the clustering approach differentiated, without using external information, between types of vegetation (light or dark vegetation; wetlands, shrubs or trees), as well as agricultural areas in the urban ecosystem, each with distinct relevance for human health and wellbeing and urban sustainability. Without clear designation of urban green spaces, which were captured by the *Vegetation* clusters, areas frequently succumb to development pressures. In our work, across all city boundaries analyzed, but especially in Accra and Dakar, the only large lush and dense green spaces within metropolitan areas (as captured by the clusters) were those officially marked as parks or forests. The conservation of these urban green spaces is important not only for recreation and mental well-being, but also for combating environmental challenges such as urban heat islands ^54,55^ and in some cases reducing the risk of flooding ^61–64^. The clustering method further identified small patches of vegetation and agriculture in Dar es Salaam and Kigali. Such areas can promote urban biodiversity and improve the living standards of the residents as urban green spaces as mechanisms to enhance food security ^65–67^. With regular availability of satellite images, our approach can assist in monitoring changes in urban green spaces or urban agriculture in relation to other urban environments, over time.

### 4.3 Strengths and limitations

To our knowledge, this work represents the first use of unsupervised deep learning techniques and satellite images to systematically capture and compare urban morphology across multiple African cities. In contrast to studies focusing on one or two discrete characteristics (e.g., roads or green space) or single-city analyses, this approach unifies multiple properties of the urban environment, offering a new lens through which to examine and track complex urban contexts. The phenotypic clusters derived here allow learning across cities to inform planning and policy interventions, and support comparative urban research, especially in regions with limited availability of ground measurement data.

An advantage of the unsupervised approach is that it can easily be extended to additional cities, unveiling both shared and unique characteristics of their natural and built environments. Its generalizability lies in its reliance solely on high-resolution satellite imagery, making it particularly suitable for regions where traditional data are limited. While differences in infrastructure, topography, and land cover may affect the specific clustering outcomes, the method’s adaptability allows it to accommodate regional variability by adjusting cluster granularity and training data coverage. Expanding the application of this approach to a broader and more diverse set of cities would be a promising future direction, offering the potential to test its robustness and enhance its relevance for cross-regional urban analysis. Similarly, this framework is adaptable for longitudinal assessments using satellite images, enabling the monitoring of urban change as different urban phenotypes expand or replace one another over time. In addition, the approach offers flexibility in terms of hierarchical clustering. For example, at the global scale, image representations may be learned together but clustered at a regional or city level. Adjusting this method for use in different cities requires careful deliberation regarding the number of clusters, which must correspond to the local environmental context and the specific objectives. A low number of clusters (K) aids in distinguishing between the built and natural environment, whereas an increased number emphasizes more specialized clusters that capture more diverse environments, possibly at the expense of interpretability. Additionally, a comparison to ESA land cover maps ^83^ reported in the Supplementary Materials showed that the cluster analysis detected patterns not represented in traditional land cover data. It also provided additional detail and sub-groups within specific land cover classes, such as built-up areas.

The main limitations of the study are related to spatial and temporal data availability. In particular, the Maxar satellite images used for the four cities were acquired between 2018 and 2022 and were composites assembled from multiple acquisition dates within one year. This was necessary to achieve full city coverage and minimize cloud cover, as single-date images often lacked sufficient visibility. Notably, Accra’s images are from 2018–2019, while those of were captured between 2020 and 2022. Although only one composite image was used per city, each represents a specific point in time, making the analysis sensitive to seasonal variation. Differences in acquisition dates and seasonal variations can particularly affect vegetation-related clusters, as vegetation appearance vary seasonally. For example, green spaces or agriculture identified during wetter seasons might appear denser compared to drier periods, potentially affecting interpretations of the resulting clusters. Access to multiple images in different seasons and meteorological conditions would allow examining the influence of such factors on visual markers of urban environment and the resultant phenotypes. Incorporating multi-temporal imagery would also make it possible to capture intra-annual variations in vegetation, land use, and surface characteristics, allowing the detection of seasonal urban transformations. This represents an important direction for future research.

The number of external variables used for interpretation of clusters was limited. Further data on the built and natural environment, such as on building height or agricultural land, might improve interpretation. Moreover, some of the external datasets used for cluster interpretation (i.e., WorldPop datasets) are available at a coarser resolution compared to the high-resolution satellite image tiles. This mismatch may obscure fine-scale relationships or introduce errors when validating local urban patterns. A further limitation is the availability and cost of obtaining high-resolution satellite imagery from commercial sources, as well as increased computational demands compared to traditional pixel-level analysis. However, our framework is able to capture more detailed information beyond what is captured by one pixel. Additionally, although the training process may be costly in terms of time and computing power, the model is highly efficient during inference and clustering. Computational costs mainly arise from pre-processing of the high-resolution images as well as running the image representation part of the framework (time and memory). Processing typically scales linearly with data volume, but more efficient and lower-cost computational resources, such as cloud platforms and access to GPUs, is rapidly reducing these barriers. Thus, the framework can easily be applied to additional satellite images for a near real-time analysis of urban environments.

## 5 Conclusions

While there are complexities and diversities in cities in SSA, we found that they have key environmental similarities, visible in high-resolution satellite images, which arise from their shared histories and recent accelerated urbanization. These similarities are complemented by unique features, many related to differences in the local geographical and climatic conditions, colonial past and urban policies that differentiate each city’s growth. This analysis has shown that unsupervised clustering of satellite images can help to elucidate complex environmental phenotypes in cities in SSA that are intuitive and interpretable, and can be used across a wide range of settings and applications, offering a robust and flexible tool for studying urban areas.

## Supplementary Material

Supporting Information

## Figures and Tables

**Table 1:** Comparison of MAD scores per city and approach.

Approach	Accra	Dakar	DeS	Kigali	All cities
**A**	2.0203	2.374	2.4905	2.3393	2.2021
**B**	**1.9235**	1.8118	2.2217	1.9675	1.6944
**C**	1.9709	**1.7357**	**2.0411**	**1.9637**	**1.6125**

Bold text indicates the best scores for each city. DeS: Dar es Salaam.

**Table 2:** Accuracy scores for city-specific classifiers.

City	Accuracy
Accra	0.6484
Dakar	0.6401
Dar es Salaam	0.7541
Kigali	0.5106

Accuracy scores of the XGBoost classifiers trained on urban characteristics to predict specific cluster membership for each city.

**Table 3:** Cluster super-groups and names across cities.

Cluster super- groups	Accra (•)	Dakar (▯)	Dar es Salaam (■)	Kigali (★)
**Vegetation**	**Veg1:** Light vegetation (7%)	**Veg1:** Shrubs and unpaved roads (8%)	**Veg1:** Vegetation and unpaved roads (6%)	**Veg1:** Shrubs (9%)
	**Veg2:** Dark dense vegetation (6%)	**Veg2:** Trees and bushy landscape (6%)	**Veg2:** Medium vegetation (7%)	**Veg2:** Wetlands (4%)
			**Veg3:** Dark dense vegetation (3%)	**Veg3:** Dark dense vegetation (6%)
**Agriculture**		**Ag1:** Agriculture (13%)	**Ag1:** Agriculture and sparsely populated areas (9%)	**Ag1:** Agriculture and sparsely populated areas (15%)
			**Ag2:** Agriculture (8%)	**Ag2:** Trees and agriculture (9%)
**Water**	**Water:** Water (2%)			
**Empty land**	**Empty1:** Empty land (10%)	**Empty1:** Sand and empty land (8%)		
**Densely populated**	**Dp1:** Densely populated, low building orientation (19%)	**Dp1:** Densely populated, low building orientation (7%)	**Dp1:** Densely populated, low building orientation (15%)	**Dp1:** Densely populated areas (23%)
	**Dp2:** Densely populated, med. Building orientation (22%)	**Dp2:** Densely populated, med. Building orientation (8%)	**Dp2:** Densely populated, high building orientation (12%)	
	**Dp3:** Densely populated, high building orientation (13%)	**Dp3:** Densely populated, high building orientation (5%)		
**Other mixed environments**	**Mix1:** Sparse-moderately populated areas and other mixed environments (21%)	**Mix1:** Sparse-moderately populated areas and other mixed environments (44%)	**Mix1:** Sparse-moderately populated areas and other mixed environments (41%)	**Mix1:** Other mixed environments, sparse-moderately populated (29%)
				**Mix2:** Mixed environment, sparsely populated; sloped area (5%)

The table lists all clusters across cities, the clusters are grouped into cluster categories such as vegetation and densely populated areas. The cluster names are used in Fig. 5 for identification. The percentages represent the proportion of each cluster group in the respective city.

## Data Availability

The satellite images of Dakar, Dar es Salaam and Kigali were purchased with a license that does not allow the publication of the images. The satellite image of Accra (and similar images) is available through the Maxar Open Data program via https://www.maxar.com/open-data. All external data used for the analysis are openly available and data sources are listed in the data table (Table 1). City maps with cluster classification and code will be made available on the Pathways to Equitable Healthy Cities research collaboration website (https://equitablehealthycities.org/data-download/) upon publication of the paper.
