# Supplementary material for: Unsupervised deep clustering of high-resolution satellite imagery reveals phenotypes of urban development in Sub-Saharan Africa
Source: Sci Total Environ. Author manuscript; Available in PMC 2025 Jul 20. (PMC7617845; doi:10.1016/j.scitotenv.2025.179739)
Supplement: Supporting Information [file EMS206337-supplement-Supporting_Information.pdf]

Supplementary Materials for

Unsupervised deep clustering of high-resolution satellite imagery reveals phenotypes of urban development in Sub-Saharan Africa

A Barbara Metzler et al.

*Corresponding author email: majid.ezzati@imperial.ac.uk

This PDF file includes:

Supplementary Text

Figs. S1 to S14

Tables S1 to S4

Supplementary Text

Study cities

Accra

The administrative, economic and political center of Ghana has a population of 5 million residents and is expected to grow to 9.6 million residents by 2050 (1). Accra is a coastal city located in West Africa and experiences a tropical savanna climate with distinct dry and wet seasons. Accra has a rich history, with evidence of human habitation dating back to the 14th century. It played a crucial role in the trans-Atlantic slave trade and later became a hub for British colonial administration. Today, Accra is a major hub for commerce and industry, and hosts numerous national and multinational entities. Its development trajectory reflects ongoing urban expansion, infrastructural improvements, and the growth of its financial and technology sectors (2,3).

Dakar

The administrative and economic capital has a population of about 3.5 million people that is expected to grow to 8.5 million in 2050 (1). Dakar is located on a peninsula surrounded by 70 kilometers of coastline and has a tropical arid climate. Dakar has historically been a key port and a major center for trade and commerce. The urban development of Dakar, influenced by its colonial past, has a blend of traditional African and European architectural styles (4,5). Currently, Dakar serves as Senegal's economic epicenter, housing major financial institutions, corporate headquarters, and a rapidly expanding telecommunications sector. Its prominence is further reinforced by the growth of business tourism and the vibrancy of the informal economy, particularly in trade, transportation, and construction, solidifying its role as a central hub for commerce and development (6).

Dar es Salaam

The former capital and largest city in Tanzania has about 7 million inhabitants today and is projected to grow to 16 million by 2050, making it the first megacity in East Africa (1). Dar es Salaam is the major port city situated on the coastline of East Africa. It is located south of the equator and has a tropical hot climate throughout the year. The port facilitates trade and commerce, handling a significant part of the country's import and export activities. Dar es Salaam's urban landscape bears the imprints of its colonial past and retains a mix of German, British and Arabic architecture, intermixed with a range of building types, such as the typical single-story, detached, six-room Swahili houses as well as high-density high-rise buildings (7,8). Today, Dar es Salaam is a regional center for commerce and industry, hosting multinational corporations, a growing manufacturing sector, and informal economies, which has created increased demand for housing and transport (9).

Kigali

The capital and largest city of Rwanda has 1.6 million inhabitants, having increased from just 6,000 inhabitants after its independence in 1962 (10). The city is expected to grow to 3.8 million inhabitants by 2050 (11). Kigali is located near the center of Rwanda, a landlocked country in East Africa. Its urban landscape has been shaped more by the impacts of war and genocide than by its colonial history, with many buildings suffering heavy damage or demolition during the civil war in 1994. Consequently, much of the city has been rebuilt, with modern office buildings, roads, and other infrastructure now defining its skyline (12). In the post-genocide era, Kigali has undergone rapid modernization, with significant investments in commercial development, quality infrastructure, and industrial growth, particularly through the Kigali Special Economic Zone. Known as one of Africa’s cleanest cities, Kigali aims to position itself as a regional hub for tourism, commerce, and innovation (13).

Sensitivity to cluster number

The sensitivity analysis to cluster number revealed that there were a few clusters that emerged early, and stayed stable over changing values for K. Fig. S9 shows the Calinski-Harabasz (CH) and silhouette scores across changing cluster number (K). The image representations form dense clusters in the feature space, resulting in a decrease of the Calinski-Harabasz (CH) score as the number of clusters (K) increases. This suggests that adding more clusters does not significantly improve the separation between groups of similar images. Nevertheless, across all cities, the curve started to flatten between K=5 and K=8. Simultaneously, there was an upward trajectory in the silhouette score with increasing K, following an initial descent around K=3.

Overall, although larger cluster numbers yield more detailed results, they also pose a challenge when it comes to interpretation. This sensitivity analysis suggests that for all cities, a value of K=8 (or similar) struck an optimal balance. It provided sufficiently granular clustering to accurately capture the structure of the data while remaining comprehensible and analyzable by researchers. This balance is crucial as it ensures that the technical aspects of the analysis do not overshadow the interpretability of the results, thus making the data actionable for decision-makers and urban planners.

Choice of learning rate

We further used the Median Absolute Deviation (MAD) score (as described in 6.4.1 Model considerations) to pick the optimal learning rate from a set of different learning rates (0.01, 0.001, 0.0001 and 0.00001). Upon investigating the outcomes of Approach A with varying cluster numbers (K = [8,12,16]), we observed that a learning rate of 0.0001 consistently yielded

the lowest MAD scores. This learning rate mirrors the optimal rate identified in the single city clustering research, as discussed in Metzler et al. (30). For Approach B, however, the learning rate necessitated adjustments on a city-by-city basis. For Approach B, the learning rate was adjusted per city and ranged widely. For Approach C, a learning rate of 0.00001 was best. All optimal learning rates are listed in Table S1.

Comparison to ESA land cover maps

In addition to interpretation of our clusters using external datasets (i.e. datasets not involved in cluster formation) on the built environment (roads and buildings), population density, water and vegetation, we compared the clusters to the 2021 world cover dataset by the European Space Agency (ESA) (71). We downloaded the land cover maps of the countries where our four cities are located, cut out the city shapefiles and re-projected the maps into the same coordinate reference system. We then spatially joined the datasets for every city. To visualize which land cover labels are most prevalent within each cluster, we created co-occurrence plots normalized by cluster as seen in Fig. S11. The clusters and their naming and correspond to the cluster maps in Fig. S3.

There was a moderate overlap between certain clusters and ESA land cover maps. In general, the densely populated areas clusters overlapped mostly with the built-up land cover class for all cities. In general, the clusters picked up patterns that were not represented in the land cover data and added further detail and sub-groups to specific land cover classes, including discerning different levels of building density and building orientation. We also found that in some cities, two different ESA land cover classes appeared visually similar, particularly the *Tree-covered area* and *Shrub-covered area* classes. Examples of these tiles are shown in Fig. S12. The plotted tiles were randomly sampled from a shuffled list of tiles assigned to each label. In

Kigali, the co-occurrence map (Fig. S11) revealed that much of the *Dark dense vegetation* cluster was labeled as *Shrubs* in the ESA land cover data. However, most of these tiles did not resemble typical shrubland; instead, they appeared significantly lusher and greener (Fig. S13). This was also very similar to the analysis in Accra, where a large proportion of the *Dark dense vegetation* cluster was labeled as *Shrubs* by the ESA land cover maps. Fig. S9 depicts randomly selected tiles that were assigned to the *Dark dense vegetation* cluster that visually do not indicate any shrub-like areas.

A further example of Dakar is shown in Fig. S14. A large part of the S*and and Empty land* cluster was labeled as *Cropland* by the ESA land cover maps. The tiles that captured *Bare areas* as defined by the ESA land cover dataset also included built environment and water, even though there is also an ESA land cover class named *Water*. The *Water* cluster in Accra was both picked up by the clustering algorithm and labelled as *Water* by the ESA land cover map. This was also highlighted by the co-occurrence plot for Accra in Fig. S11.

Fig. S1: Examples of satellite image tiles.

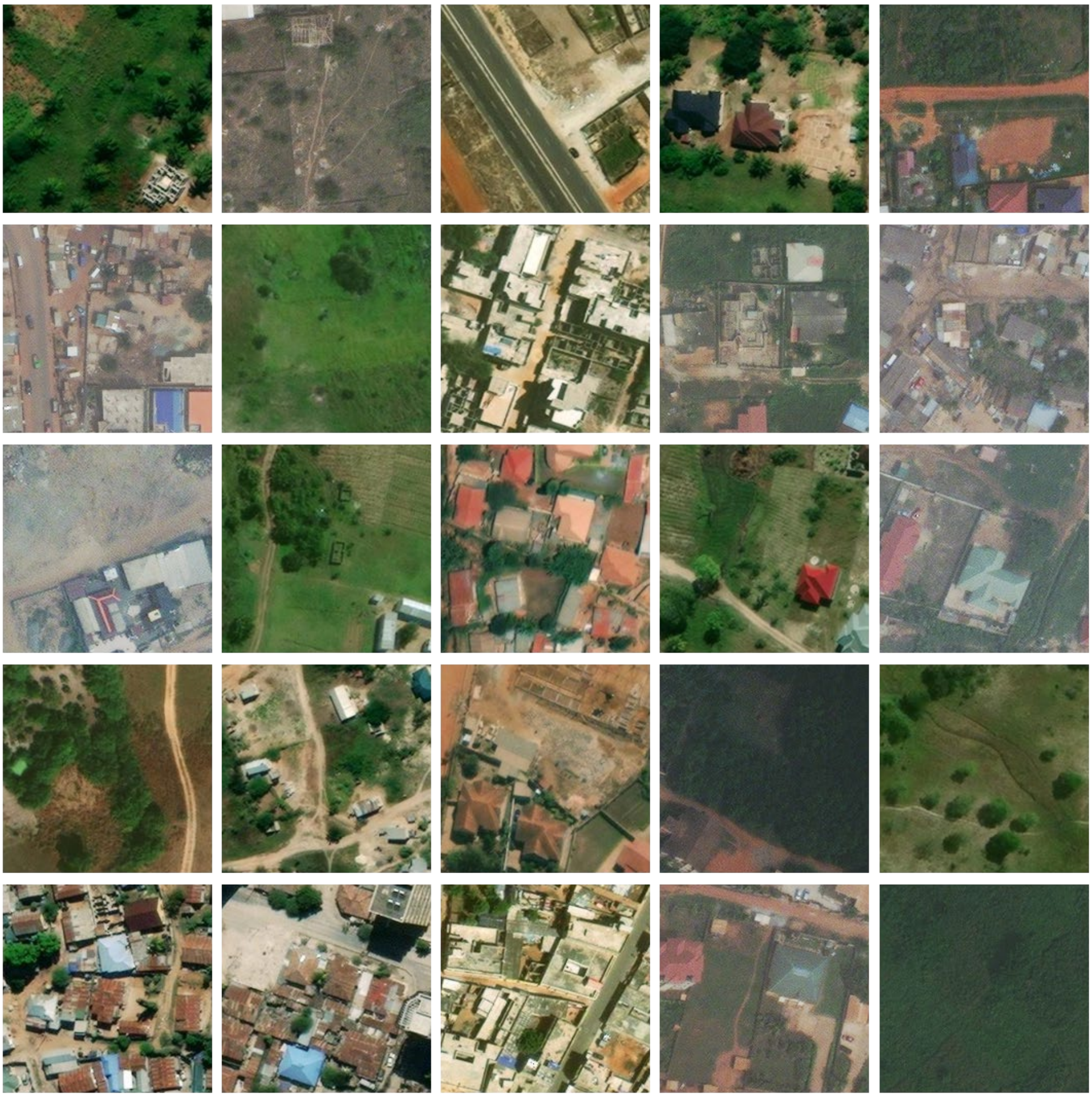

Random sample of satellite image tiles across all cities. The resolution amounts to 0.3m/pixel, which allows to see details such as roof top materials and cars. Dynamic mosaic ©2021/2 Maxar Technologies.

Fig. S2: Impact of cluster number on the clustering outcome.

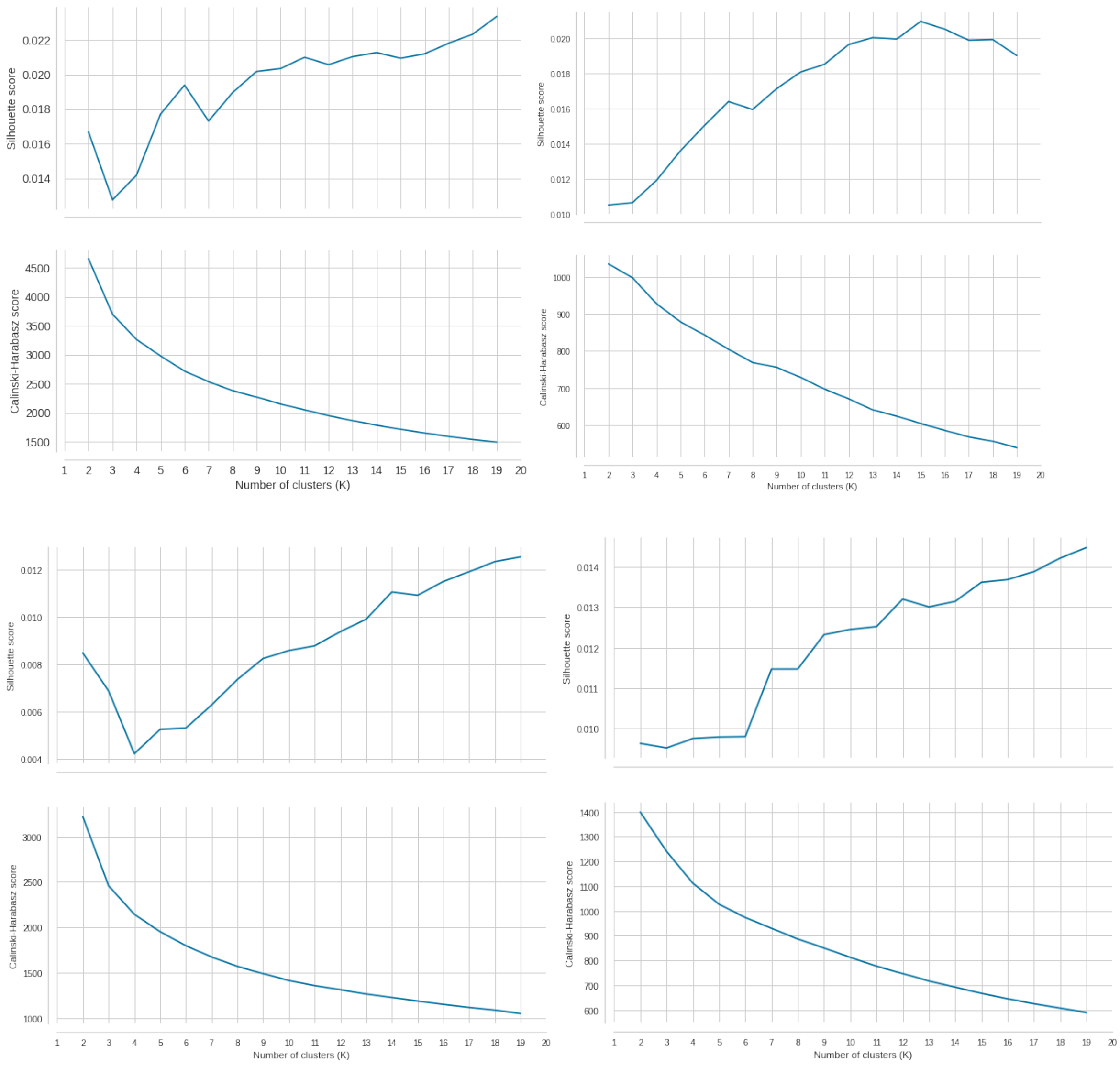

Silhouette and CH scores for K=2 to K=20 of Accra, Dakar, Dar es Salaam and Kigali. Both scores evaluate the cluster cohesion and separation of clusters for various K values, but neither method yielded a unique and definitive choice for the best number of clusters.

Fig. S3: Map of image-driven clusters in Accra, Dakar, Dar es Salaam and Kigali.

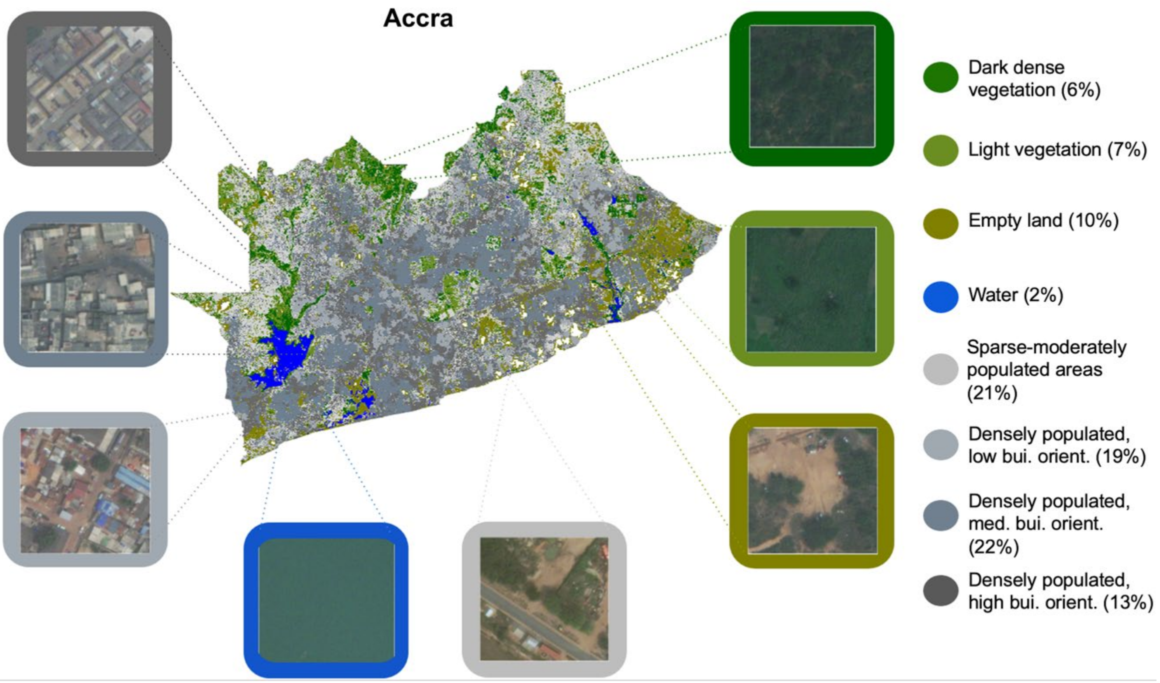

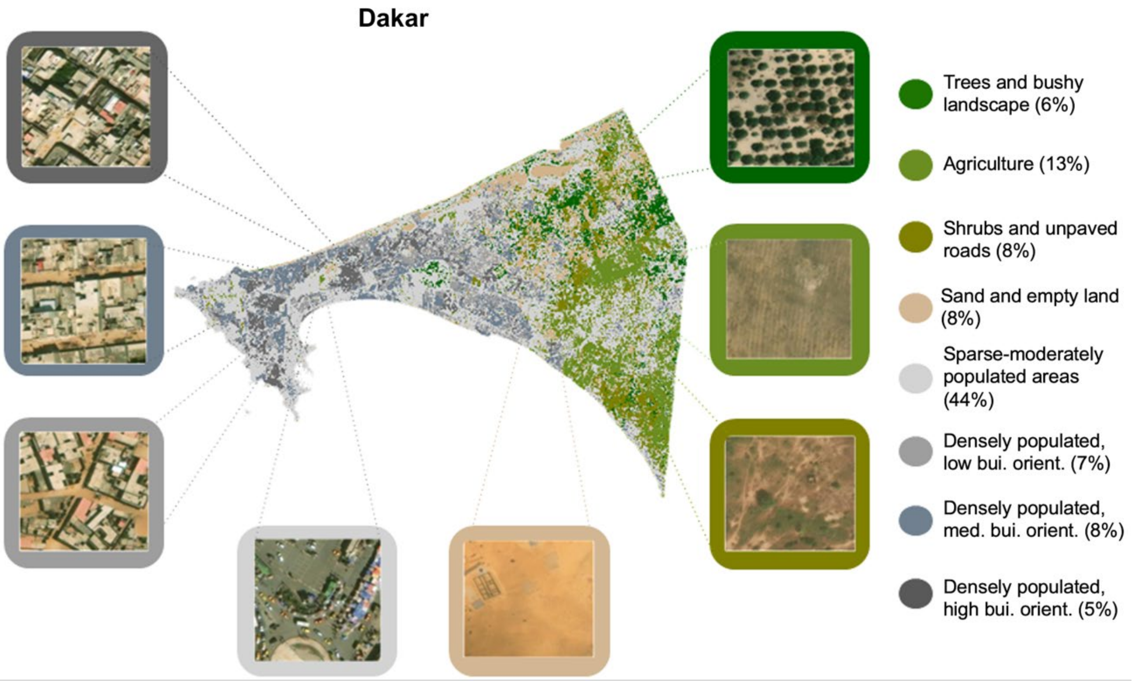

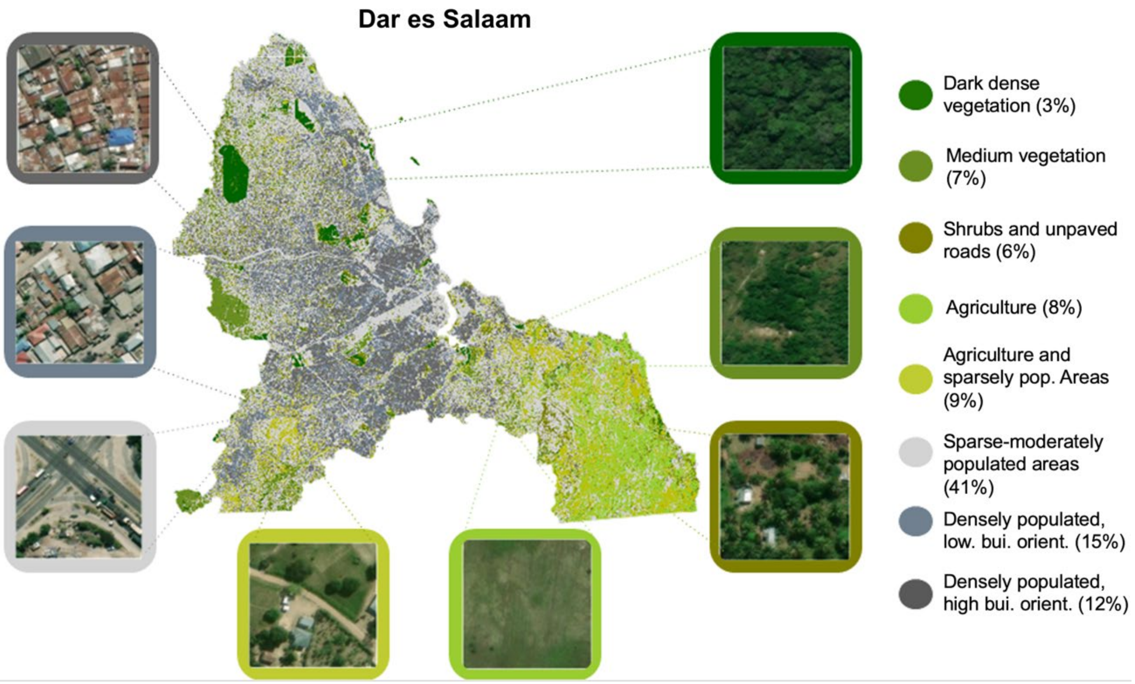

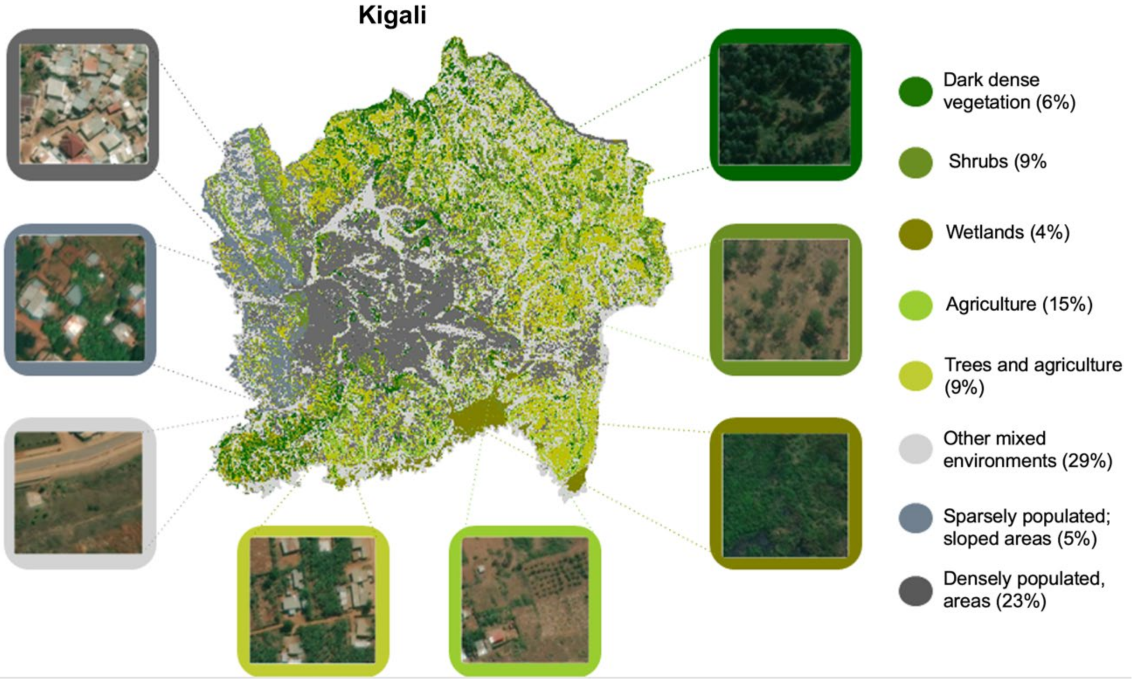

Figs. S3A to S3D show the map of image-driven clusters and example image tiles assigned to one of eight clusters for every city. Each cluster uses a consistent color, which was chosen based on the super-group that each cluster falls in. The cluster colors stay consistent in the subsequent figures. Highlighted boxes provide sample tiles from each cluster, with adjacent

numbers indicating the percentage of tiles that belong to that particular cluster. Dynamic mosaic © 2022 Maxar Technologies.

Fig. S4: Example of buildings with varying building orientation.

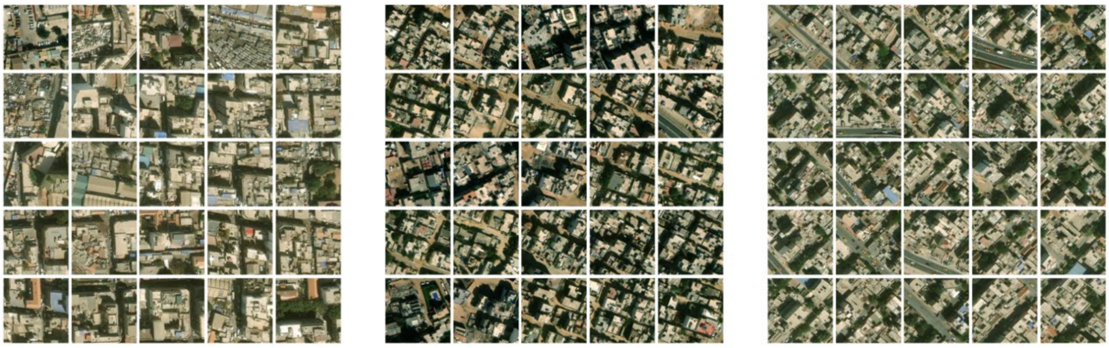

Neighborhoods in Dakar with varying building orientation ranging from low (left) to high (right). Dynamic mosaic ©2021/2 Maxar Technologies.

Fig. S5: Types of vegetation in the study cities.

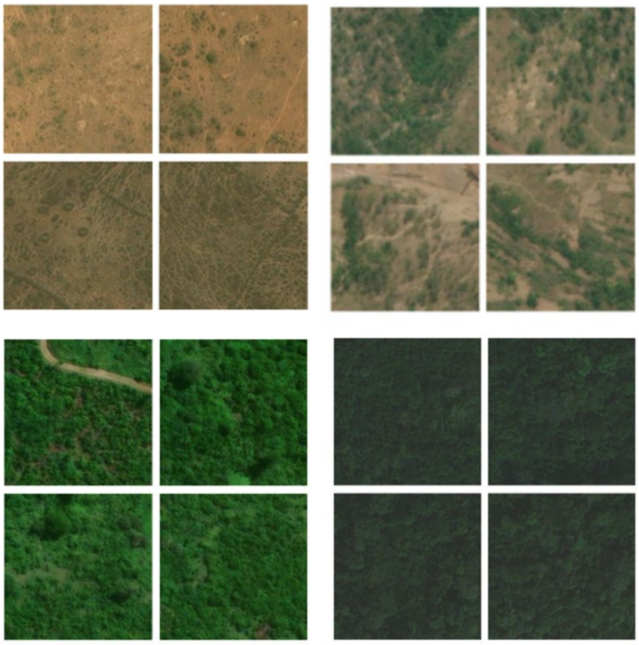

Examples of vegetation that range from shrubs in Dakar (top left) and in Kigali (top right) to dark dense vegetation in Dar es Salaam (bottom middle) and Accra (bottom right). Dynamic mosaic ©2021/2 Maxar Technologies.

Fig. S6: Examples of agriculture in the study cities.

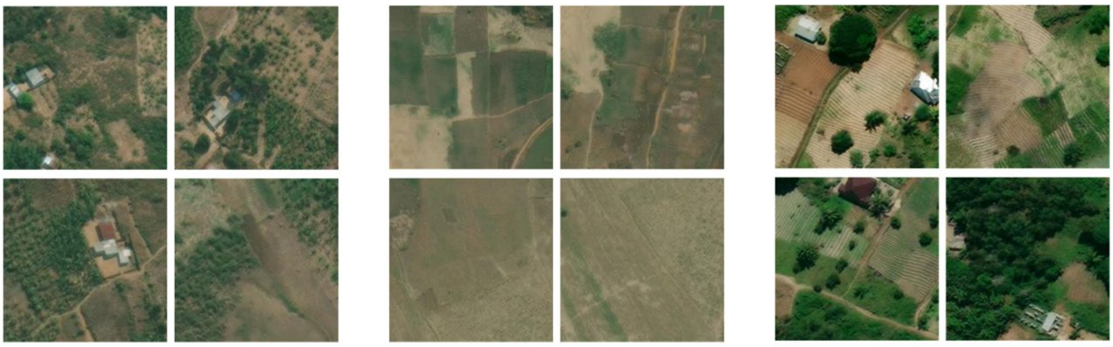

Examples of tiles capturing various types of farming and crops. Dynamic mosaic ©2021/2 Maxar Technologies.

Fig. S7: Sparse-moderately populated areas and other mixed environments.

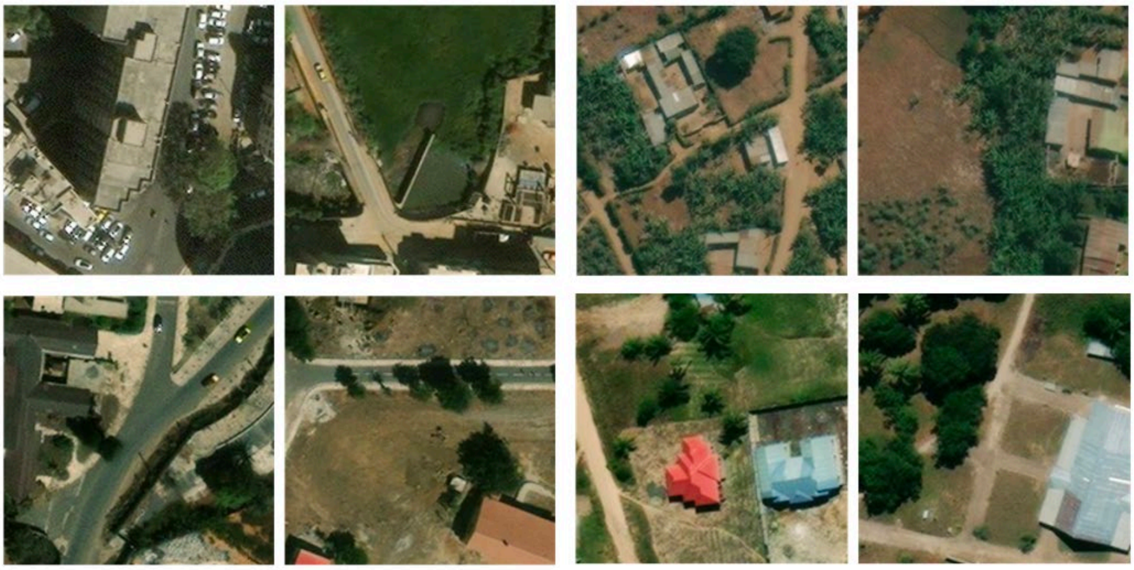

Examples of tiles that capture sparse-moderately populated areas and other mixed environments in the study cities. Dynamic mosaic ©2021/2 Maxar Technologies.

Fig. S8: Examples of tiles capturing informal settlements.

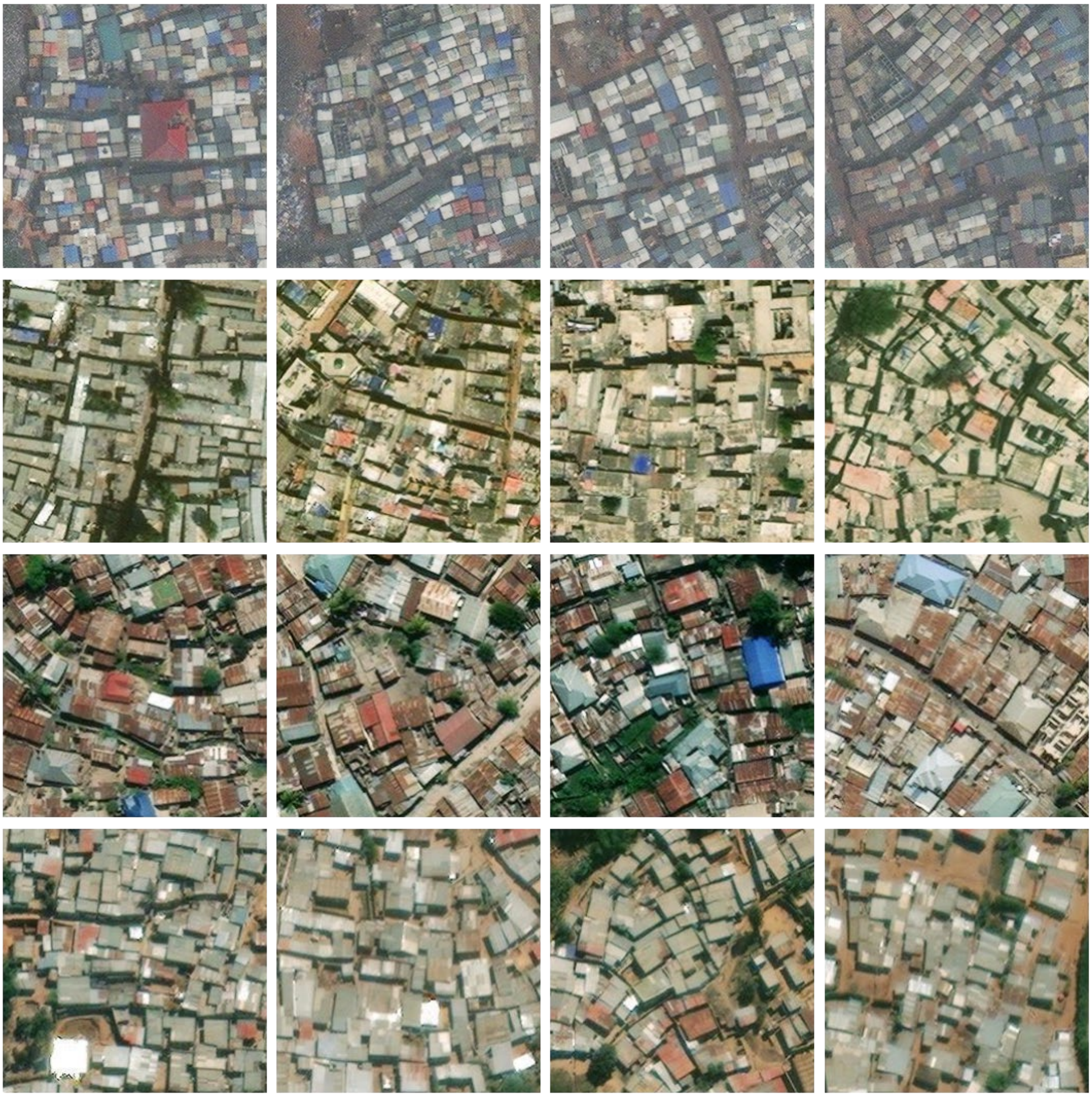

Examples of informal settlements in the study cities. Each row represents a different city: Accra, Dakar, Dar es Salaam and Kigali (from top to bottom). Dynamic mosaic ©2021/2 Maxar Technologies.

Fig. S9: Dark dense vegetation, Accra.

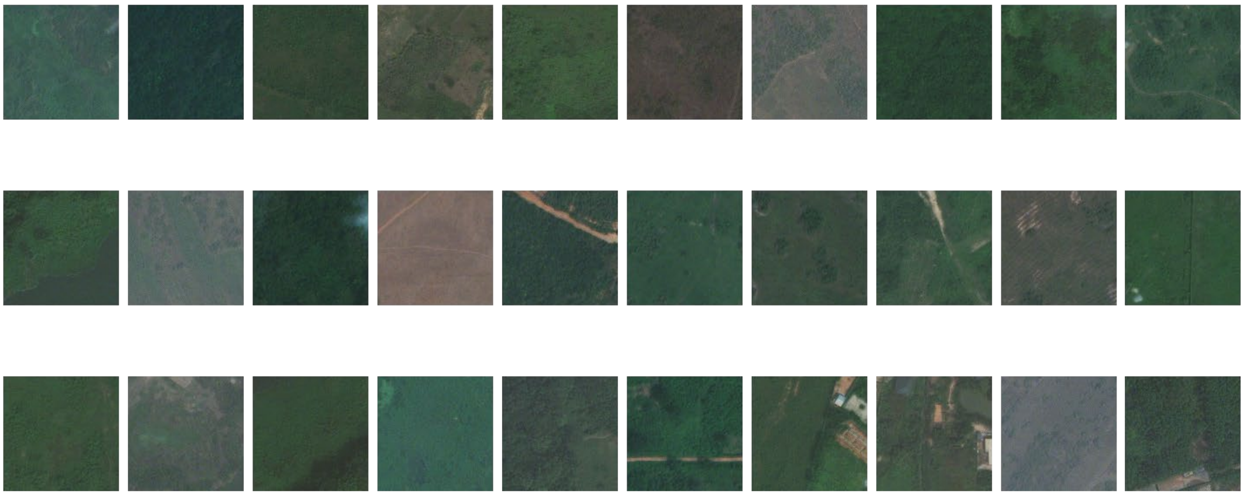

Tiles labeled as *Dark dense vegetation* cluster, Accra.

Fig. S10: Relative size of clusters per city.

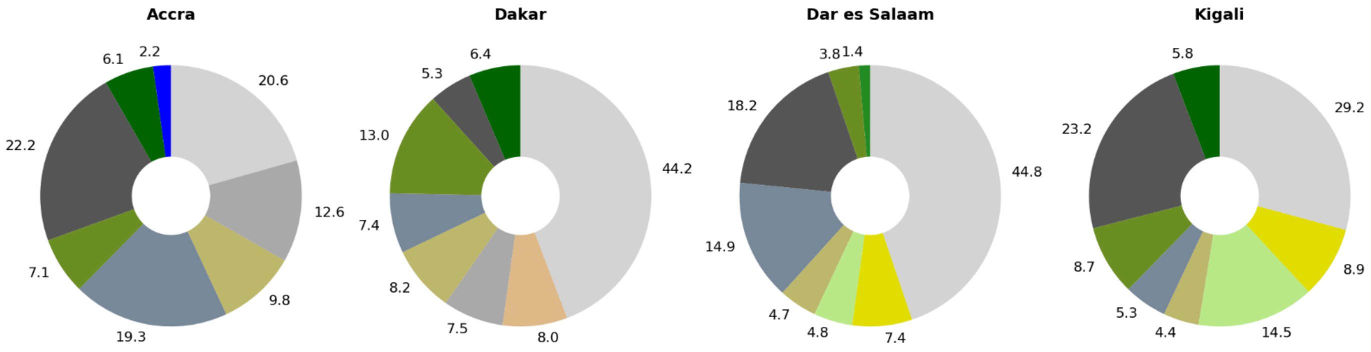

Pie charts show the relative area covered by each cluster in each city. The colors of the pie sections are the same as those used cluster maps in Fig. S3.

Fig. S11: Co-occurrence of clusters and ESA land cover classes.

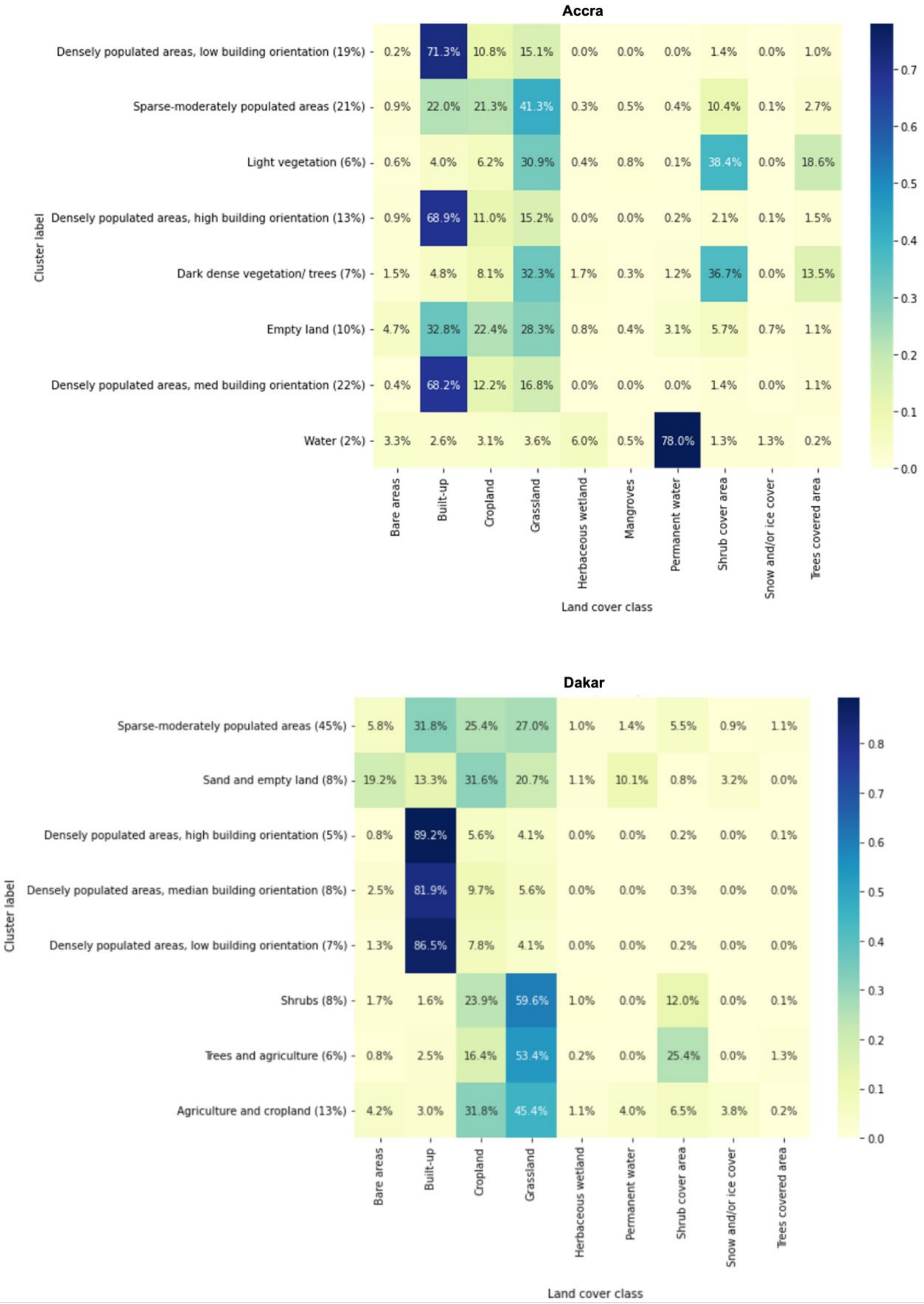

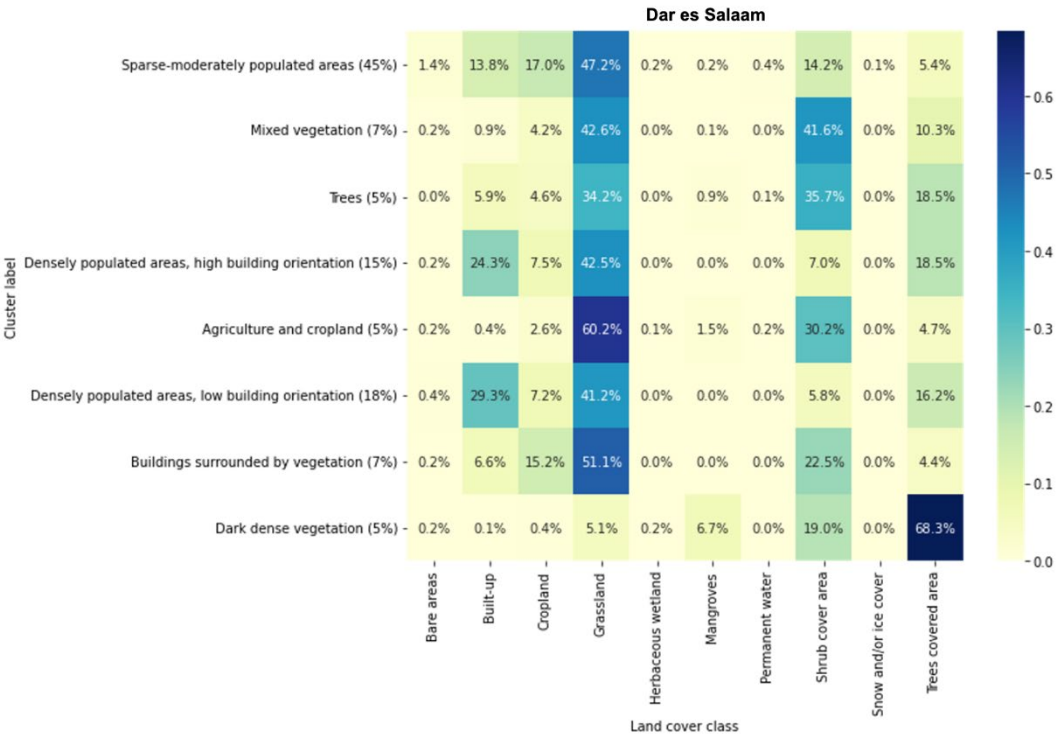

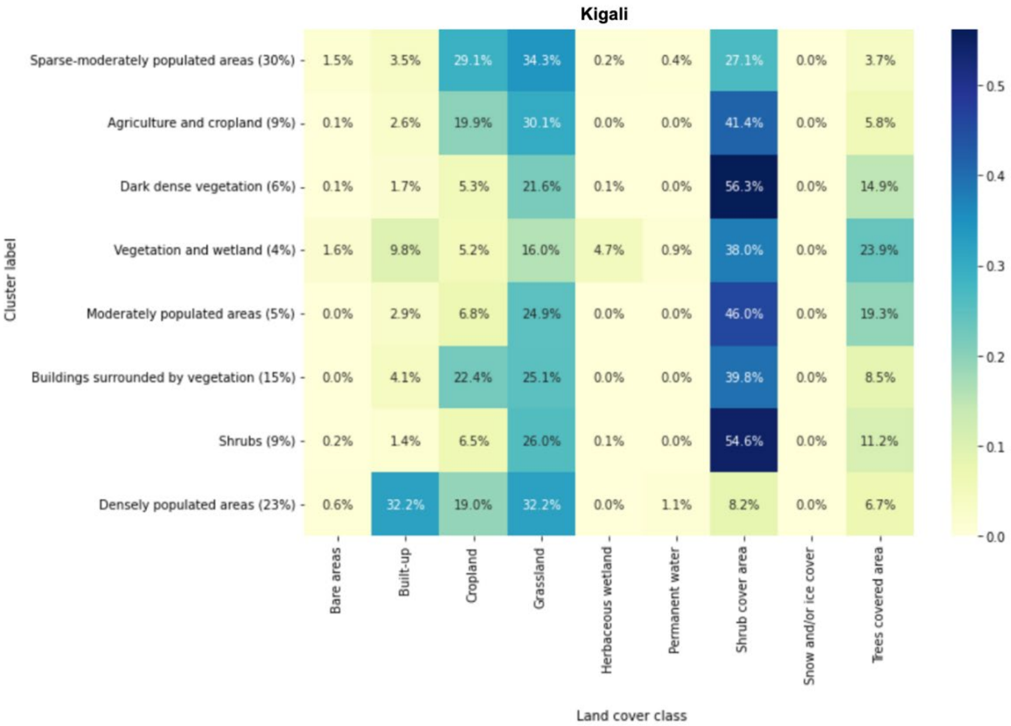

Cluster membership compared to the ESA land cover maps for all study cities. The created co-occurrence plots are normalized by cluster (row) to visualize what ESA land cover classes

co-existed with the clusters. The clusters are named by what they represent and correspond to the cluster maps in Fig. 2.

Fig. S12: Example satellite tiles of ESA land cover labels.

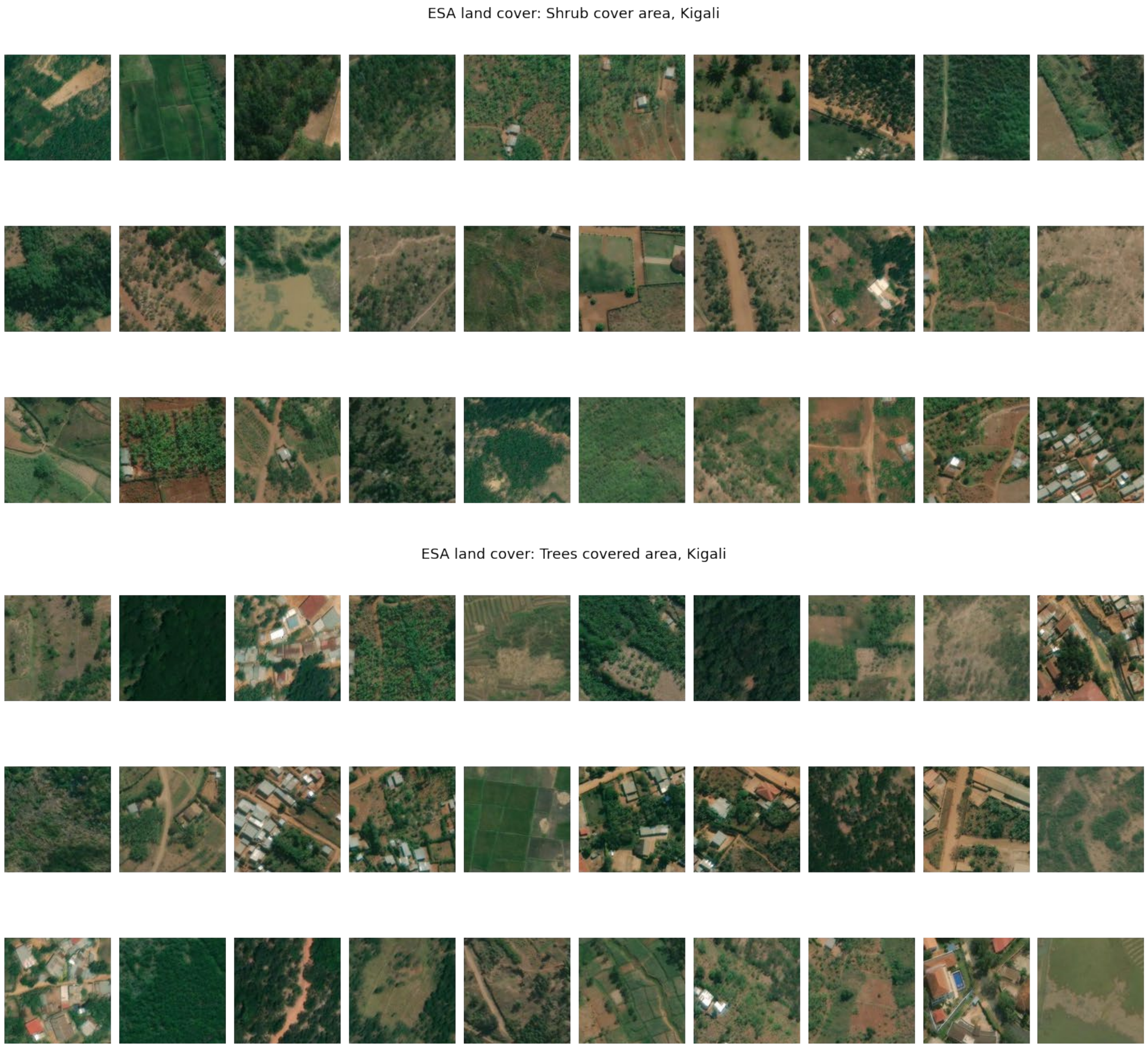

Example tiles of two different ESA land cover classes in Kigali, which are visually very similar. These tiles were sampled from a shuffled list of tiles assigned specific labels. Dynamic mosaic ©2020 Maxar Technologies.

Fig. S13: Shrubs and Dark dense vegetation clusters, Kigali.

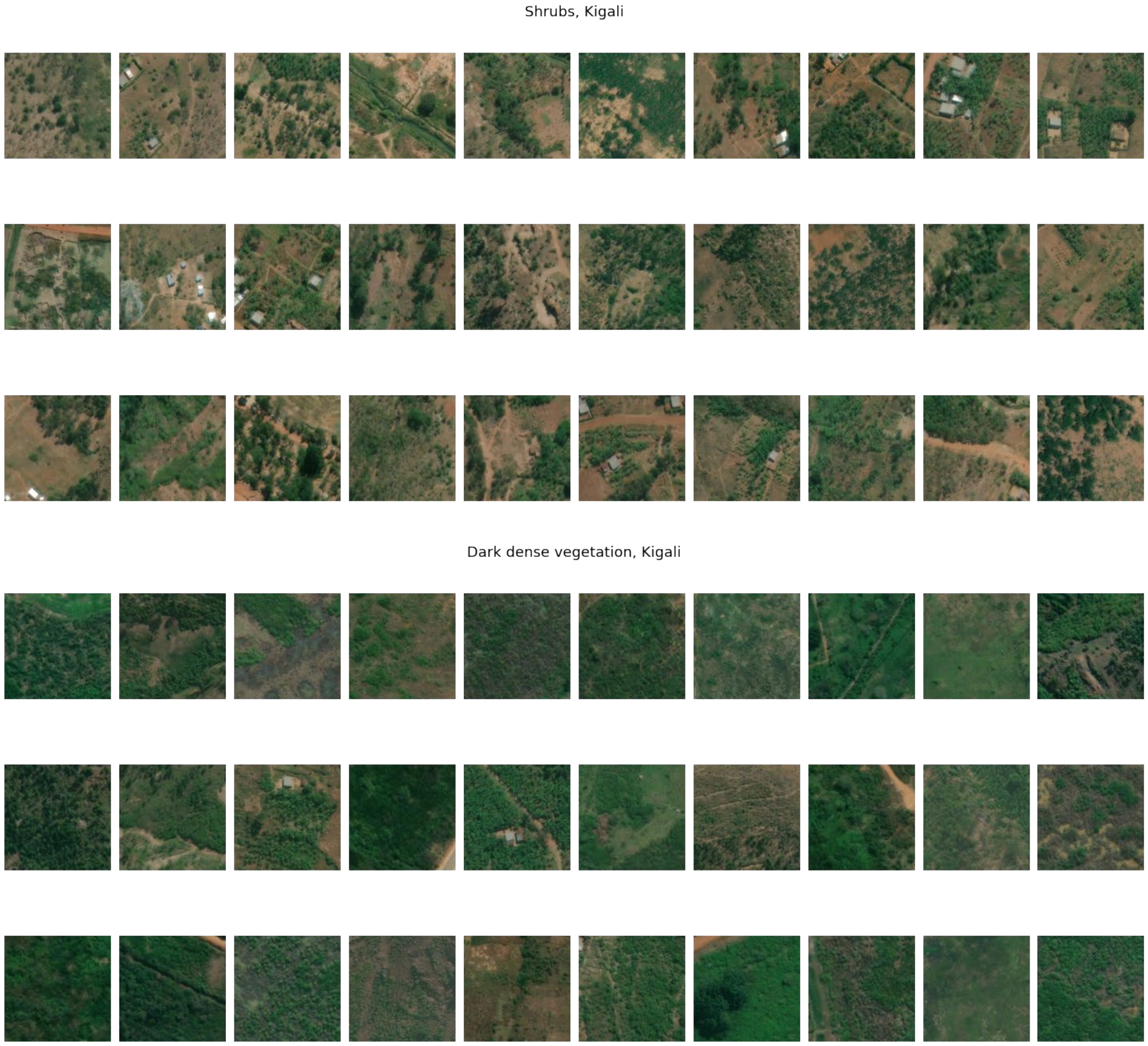

Example tiles of two different clusters in Kigali. Despite the co-occurrence map of Kigali (Fig. S11) indicating that a large part of the *Dark dense vegetation* cluster was labeled as *Shrubs* in the ESA land cover data, most tiles appear lusher and greener rather than resembling typical shrubs. Dynamic mosaic ©2020 Maxar Technologies.

Fig. S14: Sand/bare areas cluster and ESA land cover classes, Dakar.

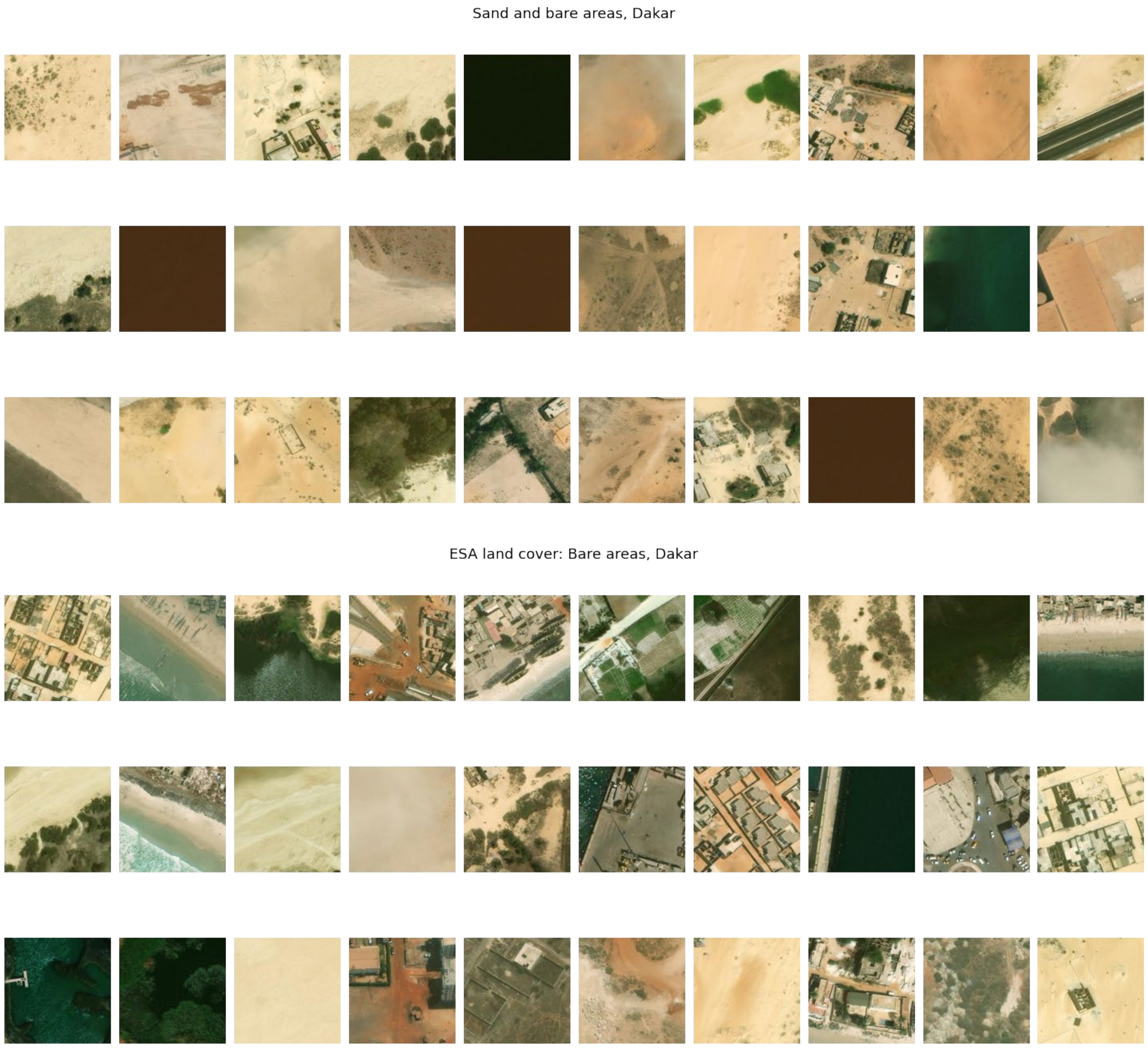

Tiles labeled as *Sand/Empty land* and water cluster and as *Bare areas* by the ESA land cover dataset, Dakar. Dynamic mosaic ©2021/2 Maxar Technologies.

Table S1: Demographic and environmental information for the study cities.

	Accra	Dakar	Dar es Salaam	Kigali
**Total population**[1]	5 million	3.5 million	7 million	1.6 million
**Area (used for analysis)**	1,312 km^2	570 km^2	1,465 km^2	750 km^2
**Average elevation**[2]	31 m	7 m	57 m	1,530 m
**Coastal/inland**	Coastal	Coastal	Coastal	Inland
**Rainfall (mm/year)**[3]	1,034 mm	508 mm	1,062 mm	1,003 mm
**Climate**	Tropical	Tropical arid	Tropical	Tropical highland
**Annual aridity**[4]	0.3	0.0652	0.2935	0.4470

[1] World Urbanization Prospects. (1)

[2] TessaDEM (14)

[3] Dataafrica.io (15)

[4] Global aridity index (16)

Table S2: Satellite images used for cluster analysis.

City	Year	Coverage (km^2)	Number of tiles
Accra	2018/19	1,312 km^2	222,420
Dakar	2021/22	570 km^2	96,627
Dar es Salaam	2021	1,465 km^2	248,375
Kiali	2020	750 km^2	127,082

Table S3: External data for cluster evaluation.

Urban characteristic	Unit	Data type	Source
Building area	m²	vector	Maxar/Ecopia.ai
Building count	integer	vector	Maxar/Ecopia.ai
Mean building size	m²	vector	Maxar/Ecopia.ai
Mean building orientation	degrees (°)	vector	Maxar/Ecopia.ai
Sum of major roads	m	vector	OSM
Sum of all roads	m	vector	OSM
Minimum distance to closest major road	m	vector	OSM
Minimum distance to closest road	m	vector	OSM
Mean population density	Estimated total number of people per grid-cell	raster (~100m/px)	WorldPop
Mean NDVI	Index (-1 to +1)	raster (~30m/px)	Landsat

[1] The building information (17) is provided in a vector format. We overlaid the vector with a grid that represents the tile size and location. For each measure, we calculated the mean value per tile. Building orientation was computed with the *momepy* package (18) as the deviation of orientation from cardinal directions; it was defined as an orientation of the longest axis of the bounding rectangle in the range 0-45 degrees.

[2] We used road information from OpenStreetMap (OSM) (19). The road information is provided in a vector format. We overlaid the vector with a grid that represents the tile size and location, and calculated statistics per tile.

[3] We used a population raster from Worldpop.org (20) with a resolution of 100 m to calculate the mean population density per tile. It was computed by vectorizing the population density raster file, overlaying it with the tile grid and calculating the mean per tile.
[4] We used Landsat satellite images (21) from a cloudless day closest to the capturing of the original satellite images, to calculate the mean NDVI value for each city and tile.

Table S4: Optimal learning rates for all approaches tested.

Approach	K	City	Learning rate
**A**	8	All cities	0.00001
	12	All cities	0.0001
	16	All cities	0.0001
**B**	8	Accra	0.0001
	8	Dakar	0.00001
	8	Dar es Salaam	0.01
	8	Kigali	0.00001
**C**	8	All cities	0.00001

As stated in above in *Choice of learning rate,* for each model, we tested a set of different learning rates (0.01, 0.001. 0.0001, and 0.00001) and chose the learning rate with the lowest Median Absolute Deviation (MAD) score to present the results.

References

1. United Nations, Department of Economic and Social Affairs, Population Division. World Urbanization Prospects: The 2018 Revision [Internet]. New York: United Nations; 2019. Available from: https://population.un.org/wup/Publications/Files/WUP2018-Report.pdf
2. Weeks JR, Hill A, Stow D, Getis A, Fugate D. Can we spot a neighborhood from the air? Defining neighborhood structure in Accra, Ghana. GeoJournal. 2007;69(1–2):9–22.
3. Abdulai AG. Accra: City report [Internet]. Manchester: African Cities Research Consortium, The University of Manchester; 2024. Report No.: ACRC Working Paper 2024-22. Available from: www.african-cities.org
4. Le Pape M. Sinou, Alain. - Comptoirs et villes coloniales du Sénégal. Saint-Louis, Gorée, Dakar. Cah DÉtudes Afr. 1993;33(132):682–3.
5. Njoh A. Planning Power: Town Planning and Social Control in Colonial Africa. London: UCL Press; 2006. 272 p.
6. Dakar [Internet]. [cited 2025 Jan 23]. Available from: https://urbanresiliencehub.org/city-economy/dakar/
7. Peter LL, Yang Y. Urban planning historical review of master plans and the way towards a sustainable city: Dar es Salaam, Tanzania. Front Archit Res. 2019 Sep 1;8(3):359–77.
8. Lupala J. Urban types in rapidly urbanising cities: analysis of formal and informal settlements in Dar es salaam. 2002.
9. Kombe WJ, Muheirwe F. Dar es Salaam: City report [Internet]. Manchester: African Cities Research Consortium, The University of Manchester; 2024. Report No.: ACRC Working Paper 2024-23. Available from: www.african-cities.org
10. Manirakiza V, Mugabe L, Nsabimana A, Nzayirambaho M. City Profile: Kigali, Rwanda. Environ Urban ASIA. 2019 Sep 1;10(2):290–307.

11. Kigali City Masterplan 2050 | Department of Economic and Social Affairs [Internet]. [cited 2023 Nov 9]. Available from: https://sdgs.un.org/partnerships/kigali-city-masterplan-2050
12. Oxford Bibliographies [Internet]. 2020 [cited 2023 Nov 9]. Kigali. Available from: https://www.oxfordbibliographies.com/display/document/obo-9780190922481/obo-9780190922481-0045.xml
13. Manirakiza V, Mugabe L, Nsabimana A, Nzayirambaho M. City Profile: Kigali, Rwanda. Environ Urban ASIA. 2019 Sep 1;10(2):290–307.
14. TessaDEM • Near-global 30-meter Digital Elevation Model (DEM) [Internet]. [cited 2024 Aug 6]. Available from: https://tessadem.com/
15. International Food Policy Research Institute (IFPRI), Datawheel. dataafrica.io. 2017 [cited 2024 Aug 6]. Data Africa. Available from: https://dataafrica.io/about#citation
16. Zomer RJ, Xu J, Trabucco A. Version 3 of the Global Aridity Index and Potential Evapotranspiration Database. Sci Data. 2022 Jul 15;9(1):409.
17. Hallas M. Mapping Africa: How Ecopia.ai and Maxar Mapped Every Building and Road in sub-Saharan Africa Using High-Resolution Satellite Imagery. AGU Fall Meet Abstr [Internet]. 2019 Dec 1 [cited 2021 Jul 30];11. Available from: http://adsabs.harvard.edu/abs/2019AGUFMIN11D0688H
18. Fleischmann M. momepy: Urban Morphology Measuring Toolkit. J Open Source Softw. 2019;4(43):1807.
19. OpenStreetMap [Internet]. [cited 2021 Sep 21]. OpenStreetMap. Available from: https://www.openstreetmap.org/
20. WorldPop. Global 100m Population [Internet]. University of Southampton; 2018 [cited 2021 Jul 30]. Available from: https://www.worldpop.org/doi/10.5258/SOTON/WP00645

21. Earth Resources Observation And Science (EROS) Center. Collection-1 U.S. Landsat Analysis Ready Data (ARD) Level-2 Data Product [Internet]. U.S. Geological Survey; 2017 [cited 2021 Jul 30]. Available from: https://www.usgs.gov/centers/eros/science/usgs-eros-archive-landsat-archives-us-landsat-analysis-ready-data-ard-level-2
